# Human health risk assessment of pharmaceuticals in the European Vecht River

**DOI:** 10.1002/ieam.4588

**Published:** 2022-02-28

**Authors:** Daniel J. Duarte, Rik Oldenkamp, Ad M. J. Ragas

**Affiliations:** ^1^ Department of Environmental Science, Institute for Water & Wetland Research Radboud University Nijmegen Nijmegen The Netherlands; ^2^ Department of Global Health‐Amsterdam, Institute for Global Health and Development, Amsterdam UMC University of Amsterdam Amsterdam The Netherlands; ^3^ Department of Environmental Sciences, Faculty of Science Open University Heerlen The Netherlands

**Keywords:** Human health, Pharmaceutical, Risk assessment, Surface water

## Abstract

Active pharmaceutical ingredients (APIs) can reach surface waters used for drinking water extraction and recreational activities, such as swimming and fishing. The aim of the present study was to systematically assess the lifetime human health risks posed by 15 individual APIs and their mixtures occurring in the German–Dutch transboundary Vecht River. An exposure model was developed and used to assess the combined risks of oral and dermal exposure under a variety of exposure conditions. A total of 4500 API uptake values and 165 lifetime risk values were estimated for 15 and 11 APIs, respectively. Overall, the lifetime human health risks posed by the APIs and their mixtures based on modeling results were deemed acceptable under typical exposure conditions. Under very extreme environmental conditions and human behavior, API mixture risks were of potential concern while the risks of individual APIs were negligible, with a few exceptions. The antibiotic doxycycline and analgesic phenazone showed the highest and lowest risks, respectively. The study did not evaluate the potential risks caused by metabolite compounds. Recommendations for water managers are provided to help improve the accuracy and utility of human health risk assessments of pharmaceuticals. *Integr Environ Assess Manag* 2022;18:1639–1654. © 2022 The Authors. *Integrated Environmental Assessment and Management* published by Wiley Periodicals LLC on behalf of Society of Environmental Toxicology & Chemistry (SETAC).

## KEY POINTS

- Human health risks from direct toxicity associated with the lifetime exposure to pharmaceutical residues in the Vecht's River catchment were predominantly below safe limits.
- Doxycycline and diclofenac pose the highest risk yet are below the risk threshold under normal environmental conditions for most individuals.
- Human features and behavior combined with environmental conditions of varied complexity can be integrated into a relatively simple deterministic exposure model to estimate lifetime health risks of pharmaceuticals in the water environment.
- Local, regional, and national authorities can readily gain insight into the potential human health risks based on minimal information, bypassing unnecessary and laborious risk assessment.

## INTRODUCTION

Medicinal products are a cornerstone of modern society. They contain active pharmaceutical ingredients (APIs) that typically elicit potent biological activity at low concentrations. Active pharmaceutical ingredients are used for their therapeutic qualities, including reducing morbidity and mortality. Following consumption, APIs are metabolized and excreted in their parent and metabolite forms at variable fractions (Celiz et al., 2009). These forms can ultimately reach the environment, where they have been detected in a myriad of environmental matrices. In surface waters, for example, APIs have been detected in the ng/L to µg/L concentration range (aus der Beek et al., 2016). Toxicological effects in wildlife (based on field studies) caused by pharmaceutical residues at environmentally relevant concentrations have been reported (Arnold et al., 2014; Oaks et al., 2004; Sanchez et al., 2011), motivating environmental risk assessment of APIs as an active field of research and regulation.

The European Union has several statutes in place aiming to protect human health against potential adverse effects of water pollutants. Examples include the Bathing Water Directive (2006/7/EC), the Water Framework Directive (2000/60/EC), and the Drinking Water Directive (2020/2184). However, none of these directives has environmentally protective standards for APIs, and detailed guidelines to specifically assess the human health risks of APIs are lacking (EU, 2000, 2006, 2020). As a consequence, human health risks due to direct and indirect environmental exposure to APIs are rarely assessed. The few scientific studies that are available usually conclude that human health risks of environmental exposures to APIs are negligible (Cunningham et al., 2009; de Jesus Gaffney et al., 2015; de Jongh et al., 2012; Kumar et al., 2010; Roden et al., 2015). However, these studies are typically limited in scope, for example, by focusing on individual APIs, a single exposure route (e.g., ingestion) or exposure patterns that are not representative for the behavior of specific groups such as swimmers and fish consumers (Bercu et al., 2008; Christensen, 1998; Leung Ho et al., 2013; Muñoz et al., 2010; Schulman et al., 2002; Shanmugam et al., 2014; Webb, 2001). Human health risks from standard exposure situations involving single APIs are likely to be limited and site‐specific. Still, humans can be exposed to a multitude of APIs through different exposure pathways, behaviors, and concentrations that can vary substantially over space and time. Therefore, local and regional water managers may struggle with the question of whether human health is sufficiently protected.

The aim of the current paper is to present a screening approach that estimates lifelong human health risks by systematically integrating exposure routes of multiple APIs and assessing their combined effects. The approach is illustrated in a case study using concentrations of 15 APIs in the German–Dutch transboundary Vecht River. Based on the results of this study we hope to (1) find out whether the integrated human health risks resulting from direct and indirect exposure to APIs in the Vecht River can be considered acceptable, (2) inform local, regional, and (inter)national water managers by showing how an integrated human health risk assessment of APIs can be performed, and (3) propose simple alternatives for assessing the integrated human health risks of multiple APIs under data‐poor settings, making onerous and exhaustive assessments superfluous.

## DATA AND METHODS

### Vecht River

**Figure 1:**
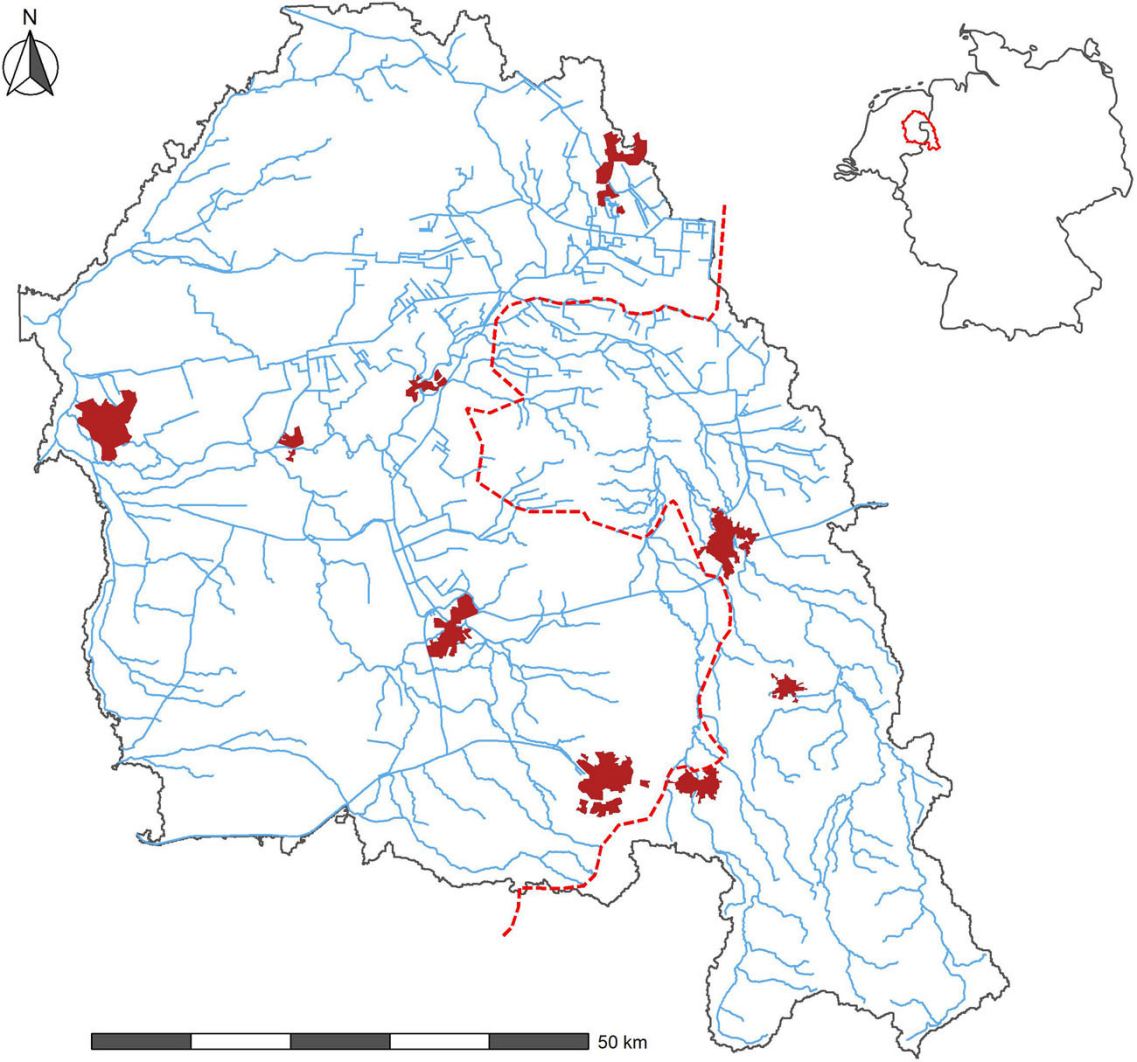
Vecht River basin. The red dashed line and the dark red closed polygons indicate the Dutch–German border and main cities, respectively

The Vecht River is a transboundary river that crosses several regions in the European Union member states of Germany and the Netherlands (Figure 1). The Vecht River is a tributary of the Dutch IJssel River with a total length of 167 km and covering a catchment area of 6100 km^2^, reaching from the northwest of Germany (160 inhabitants/km^2^) to the east of the Netherlands (260 inhabitants/km^2^). Municipal wastewater from 1.4 million inhabitants and 13 hospitals is collected by 57 sewage treatment plants and subsequently discharged into the Vecht River and its tributaries (Duarte et al., 2022; Lämmchen et al., 2021; Wöhler et al., 2020). Contributions from industrial and agricultural discharges were not characterized in this study. The area attracts numerous visitors, particularly in the Vechtdal region of the Dutch province of Overijssel. This region is actively promoted by local entities for its outdoor leisure activity opportunities, including recreational swimming and fishing, registering 2.5 million overnight stays and 90 million euros spent in 2019 (www.marketingoost.nl).

### Pharmaceuticals

Human health risks were assessed for 15 selected APIs (Table 1). This selection was made within the context of the MEDUWA‐Vecht(e) project (www.meduwa.uni-osnabrueck.de), to represent a wide range of therapeutic classes, physicochemical properties, biodegradation potential, and available ecotoxicity data. The selection includes APIs on the Watch List under the Water Framework Directive (EU, 2013; Gomez Cortes et al., 2020) (diclofenac, erythromycin, 17α‐ethinylestradiol), understudied APIs (e.g., amantadine), highly prescribed APIs (e.g., metformin, metoprolol, valsartan, diclofenac, 17α‐ethinylestradiol), and APIs with toxicity potential. Doxycycline, erythromycin, and sulfamethazine are used as veterinary medicines in the study region (Wöhler et al., 2020). Consequently, the exposure to these compounds could be underestimated due to uncertainty in the annual masses being discharged into the environment. Metabolites and transformation products (TPs) of APIs were not considered in the present study.

**Table 1:** Names, CAS numbers, ATC codes, and therapeutic classes of the 15 active pharmaceutical ingredients (APIs) assessed in the present study

API	Abbreviation	CAS RN	ATC code	Therapeutic class
Amantadine	AMA	768‐94‐5	N04BB01	Anti‐parkinson
Carbamazepine	CBZ	298‐46‐4	N03AF01	Antiepileptics
Ciprofloxacin	CIP	85721‐33‐1	J01MA02	Antibacterials
Cyclophosphamide	CYC	50‐18‐0	L01AA01	Antineoplastics
Diclofenac	DCF	15307‐86‐5	M01AB05	NSAIDs
Doxycycline	DOX	564‐25‐0	J01AA02	Antibacterials
Erythromycin	ERY	114‐07‐8	J01FA01	Antibacterials
17α‐Ethinylestradiol	EE2	57‐63‐6	G03CA01	Sex hormones
Iopamidol	IOP	60166‐93‐0	V08AB04	Contrast media
Metformin	MET	657‐24‐9	A10BA02	Antidiabetics
Metoprolol	MEP	37350‐58‐6	C07AB02	β‐Blockers
Oxazepam	OXA	604‐75‐1	N05BA04	Anxiolytics
Phenazone	PHE	60‐80‐0	N02BB01	Analgesics
Sulfamethazine	SUL	57‐68‐1	J01EB03	Antibacterials
Valsartan	VAL	137862‐53‐4	C09CA03	Angiotensin II receptor blockers

Abbreviations: ATC, Anatomical therapeutic chemical; NSAIDs, Non‐steroidal anti‐inflammatory drugs

### Exposure model

**Figure 2:**
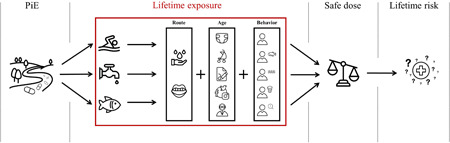
Schematic presentation of the human health risk assessment. The lifetime exposure model developed in the present study is demarcated in the red box. Three human activities were accounted for, namely, swimming, water‐drinking, and fishing. Two exposure routes were accounted for, namely, the dermal and oral routes. Five age groups were accounted for, namely, 0–1, 1–5, 5–10, 10–18, and 18–80 age groups. Five main behavioral profiles were accounted for, namely, “Average,” “Fisherman,” “Swimmer,” “Drinker,” and “Extreme” profile. PiE, pharmaceuticals in the environment.

A human lifetime exposure model (Figure 2) was created based on algorithms of a previously published model (Oldenkamp et al., 2013; Ragas & Huijbregts, 1998; Ragas et al., 2011). A detailed overview of the model's equations and parameters is presented in Table 2. The aim of this exposure model was to estimate exposure from multiple routes and quantify the systemic uptake in the human body, that is, uptake in the bloodstream. The uptake was estimated as a lifetime‐averaged daily uptake, which is ultimately compared with an internal safe dose (ISD), resulting in a hazard quotient (HQ). The ISD (Table S10) was calculated by multiplying the oral absorption fraction of an API with its safe dose for oral exposure, for example, the Acceptable Daily Intake (for threshold substances; Table 2—Equation 2) or the dose that corresponds to a 1 in 10 000 lifetime cancer risk (for genotoxic carcinogens; Dutch standard; Table 2—Equation 3). Unfortunately, human reference doses were lacking for amantadine, iopamidol, oxazepam, and sulfamethazine, implying that we could not assess their human health risks.

**Table 2:** Equations used to calculate human lifetime uptake and hazard

Equation number	Equation	Parameter	Unit	Description
(1)	$HQ=\frac{U_{t}}{ISD}$	HQ	1	Hazard quotient of a pharmaceutical
		$U_{t}$	mg/kg/day	Total uptake of a pharmaceutical in a lifetime
		ISD	mg/kg/day	Pharmaceutical internal safe dose
(2)	$ISD={\mathrm{RfD}}_{\mathrm{oral}}\times f_{\mathrm{GI}}$	ISD	mg/kg/day	Internal reference dose
		${\mathrm{RfD}}_{\mathrm{oral}}$	mg/kg/day	Oral reference dose (of threshold or nonthreshold compounds)
		$f_{\mathrm{GI}}$	%	Fraction of contaminant absorbed in the gastrointestinal tract
(3)	${\mathrm{RfD}}_{\mathrm{oral\_nt}}=\frac{ECR}{CSF_{\mathrm{oral}}}$	${\mathrm{RfD}}_{\mathrm{oral\_nt}}$	mg/kg/day	Oral reference dose (of nonthreshold compounds)
		ECR	1	Extra cancer risk in the environment
		$CSF_{\mathrm{oral}}$	mg/kg/day	Cancer slope factor via oral exposure
(4)	$U_{t}=\sum_{i=1}^{n}\left(\frac{y_{i}}{y_{\mathrm{lt}}}U_{i}\right)$	$U_{t}$	mg/kg/day	Total uptake of a pharmaceutical in a lifetime
		$U_{i}$	mg/kg/day	Total pharmaceutical uptake in age group i
		$y_{i}$	year	Number of years in an age group i
		$y_{\mathrm{lt}}\;$	year	Human lifetime expectancy (i.e., 80 years)
		*n*	‐	Number of age groups
(5)	$U_{i}=U_{\mathrm{oral},i}+U_{\mathrm{dermal},i}$	$U_{i}$	mg/kg/day	Total pharmaceutical uptake in age group i
		$U_{\mathrm{oral},i}$	mg/kg/day	Total pharmaceutical uptake via oral exposure in age group *i*
		$U_{\mathrm{dermal},i}$	mg/kg/day	Total pharmaceutical uptake via dermal exposure
(6)	$U_{\mathrm{oral}}=U_{\mathrm{os}}+U_{\mathrm{dw}}+U_{f}$	$U_{\mathrm{oral}}$	mg/kg/day	Total pharmaceutical uptake via oral exposure
		$U_{\mathrm{os}}$	mg/kg/day	Pharmaceutical uptake after water ingestion during recreational swimming
		$U_{\mathrm{dw}}$	mg/kg/day	Pharmaceutical uptake after ingestion of drinking water
		$U_{f}$	mg/kg/day	Pharmaceutical uptake after ingestion of fish
(7)	$U_{\mathrm{os}}=\frac{q_{s}\times t_{e}\times s_{e}\times f_{\mathrm{GI}}\times C_{w}}{d\times m}$	$U_{\mathrm{os}}$	mg/kg/day	Pharmaceutical uptake after water ingestion during recreational swimming
		$q_{s}$	mL/min	Rate of water swallowing while swimming
		$t_{e}$	min/event	Duration per swimming event
		$s_{e}$	events/year	Number of swimming events per year
		$f_{\mathrm{GI}}$	%	Gastrointestinal absorption fraction
		$C_{w}$	mg/mL	Pharmaceutical concentration in the swimming water
		d	days/year	Number of days in a year (365)
		m	kg	Human body weight
(8)	$U_{\mathrm{dw}}=\frac{q_{w}\times f_{\mathrm{GI}}\times C_{\mathrm{dw}}}{m}$	$U_{\mathrm{dw}}$	mg/kg/day	Pharmaceutical uptake after ingestion of drinking water
		$q_{w}$	mL/day	Amount of drinking water ingested per day
		$f_{\mathrm{GI}}$	%	Gastrointestinal absorption fraction
		$C_{\mathrm{dw}}$	mg/mL	Concentration in drinking water
		m	kg	Human body weight
(9)	$U_{f}=\frac{q_{f}\times f_{\mathrm{GI}}\times C_{f}}{m}$	$U_{f}$	mg/kg/day	Pharmaceutical uptake after ingestion of fish
		$q_{f}$	mg/day	Daily amount of fish tissue ingested
		$f_{\mathrm{GI}}$	%	Gastrointestinal absorption fraction
		$C_{f}$	mg/mg	Pharmaceutical concentration in fish tissue
		m	kg	Human body weight
(10)	$C_{f}=C_{w}\times BCF$	$C_{f}$	mg/mg	Pharmaceutical concentration in fish tissue
		$C_{w}$	mg/mL	Pharmaceutical concentration in surface water
		BCF	mL/mg	Pharmaceutical‐specific bioconcentration factor
(11)	$U_{\mathrm{dermal}}=\frac{A_{s}\times f_{s}\times k_{p}\times t_{e}\times s_{e}\times C_{w}}{d\times m}$	$U_{\mathrm{dermal}}$	mg/kg/day	Total pharmaceutical uptake via dermal exposure
		$A_{s}$	cm^2^	Human body surface area
		$f_{s}$	%	Total fraction of exposed skin during swimming
		$k_{p}$	cm/min	Skin permeability coefficient
		$t_{e}$	min/event	Duration per swimming event
		$s_{e}$	events/year	Number of swimming events per year
		$C_{w}$	mg/cm^3^	Pharmaceutical concentration in surface water
		d	days/year	Number of days in a year (365)
		m	kg	Human body weight
(12)	$A_{s}=73.31\times h^{0.725}\times m^{0.425}$	$A_{s}$	cm^2^	Human body surface area
		h	cm	Human body height
		m	kg	Human body weight
(13)	$f_{s}=1+f_{\mathrm{HSA}}\times {(S}_{f}-1)$	$f_{s}$	%	Total fraction of exposed skin during swimming
		$f_{\mathrm{HSA}}$	1	Human head‐to‐body surface area
		$S_{f}$	‐	Probability of full body submergence in a swimming event
(14)	$\log k_{p}=0.71\times \log K_{\mathrm{ow}}-0.0061\times MW-6.3$	$k_{p}$	cm/min	Pharmaceutical skin permeability coefficient
		$K_{\mathrm{ow}}$	1	Octanol–water partition coefficient
		MW	g/mol	Molecular weight of the pharmaceutical
(15)	$HI_{\mathrm{int}}=\sum_{i=1}^{n}\left(HQ_{i}\times \sum_{j\neq i}^{n}f_{\mathrm{ij}}\times M_{\mathrm{ij}}^{B_{\mathrm{ij}}\theta_{\mathrm{ij}}}\right)$	$HI_{\mathrm{int}}$	1	Interaction‐based hazard index of pharmaceutical mixture
		$HQ_{i}$	1	Hazard quotient of pharmaceutical *i*
		$f_{\mathrm{ij}}$	1	Exposure factor of the pharmaceutical pair *i* and *j*
		$M_{\mathrm{ij}}$	‐	Interaction magnitude of the pharmaceutical pair *i* and *j*
		$B_{\mathrm{ij}}$	‐	Binary weight‐of‐evidence factor of the pharmaceutical pair *i* and *j*
		$\theta_{\mathrm{ij}}$	1	Relative proportion weighting factor of the pharmaceutical pair *i* and *j*
		n	‐	Total number of pharmaceuticals in the mixture
(16)	$f_{\mathrm{ij}}=\frac{HQ_{j}}{HI_{\mathrm{add}}-HQ_{i}}$	$f_{\mathrm{ij}}$	1	Exposure factor of the pharmaceutical pair *i* and *j*
		$HQ_{i}$	1	Hazard quotient of pharmaceutical *i*
		$HQ_{j}$	1	Hazard quotient of pharmaceutical *j*
		$HI_{\mathrm{add}}$	1	Additivity‐based hazard index of pharmaceutical mixture
(17)	$HI_{\mathrm{add}}=\sum_{i=1}^{n}HQ_{i}$	$HI_{\mathrm{add}}$	1	Additivity‐based hazard index of pharmaceutical mixture
		$HQ_{i}$	1	Hazard quotient of pharmaceutical *i*
		n	‐	Total number of pharmaceuticals in the mixture
(18)	$\theta_{\mathrm{ij}}=\frac{\sqrt[{2}]{{\mathrm{HQ}}_{i}\times {\mathrm{HQ}}_{j}}}{\left({\mathrm{HQ}}_{i}+{\mathrm{HQ}}_{j}\right)\times 0.5}$	$\theta_{\mathrm{ij}}$	1	Relative proportion weighting factor of the pharmaceutical pair *i* and *j*
		$HQ_{i}$	1	Hazard quotient of pharmaceutical *i*
		$HQ_{j}$	1	Hazard quotient of pharmaceutical *j*

The lifetime‐averaged daily pharmaceutical uptake was estimated by adding the time‐weighted total uptake of five age groups (Table 2—Equation 4) that approximately represent distinct developmental stages: infant (0–1 years), toddler (1–5 years), child (5–10 years), adolescent (10–18 years), and adult (18–80 years). This subgrouping allows us to identify and allocate in more detail the fraction of pharmaceutical uptake during fundamental stages of human life. The total exposure of each age group was calculated by adding oral and dermal uptake values (Table 2—Equation 5). Human exposure to pharmaceuticals via inhalation was not included in this assessment, considering the generally very low degree of volatilization of these substances (10^−30^ to 10^−1^ mmHg at 25 °C) (Kim et al., 2021). Oral uptake of pharmaceuticals was considered to occur after (1) accidental ingestion of surface water during recreational swimming in the Vecht River, (2) consumption of Vecht‐derived drinking water, and (3) consumption of fish caught in the Vecht River (Table 2—Equations 6–10). Dermal uptake of pharmaceuticals was considered during recreational swimming in the Vecht River (Table 2—Equations 11–14). Data analysis and visualizations were performed with the statistical software R version (R Core Team, 2019) using the packages *classInt, cowplot, ggplot2, ggspatial, RColorBrewer, rgdal, rnaturalearth, scales, sf, sp, tidyverse*, and *viridis*.

### API concentrations in surface and drinking water

Table 3 presents API concentrations in Vecht River water and Vecht‐derived drinking water used in the present study. For Vecht River water, we used the mean and maximum API estimated concentrations based on human consumption as reported in our previous modeling study. For Vecht‐derived drinking water, we used measured API concentrations and their corresponding quantification limits obtained from a measurement campaign by the Dutch water company Vitens (personal communication, 1*^st^* June 2021). Since only iopamidol was actually detected in drinking water, we decided to assume either a zero concentration or a concentration equaling the quantification limit. Based on these data, we defined three concentration profiles for API concentrations in surface and drinking water:

(I) mean surface water concentrations and zero drinking water concentrations;
(II) maximum surface water concentrations and zero drinking water concentrations; and
(III) maximum surface water concentrations and drinking water concentrations equal to the analytical limit of quantification.

**Table 3:** Pharmaceutical‐specific input parameters

API	${\mathrm{RfD}}_{\mathrm{oral}}$ (mg/kg/day)	$CSF_{\mathrm{oral}}$ (mg/kg/day)	ECR(1)	$f_{\mathrm{GI}}$ (%)	$k_{p}$ (cm/min)	$K_{\mathrm{ow}}$(1)^a^	MW (g/mol)^b^	$C_{w}$ (µg/L)^c^,^d^,^e^	$C_{\mathrm{dw}}$ (µg/L)^f^	BCF (L/kg)^g^
EE2	0.000167^h^	–	–	100^i^	1.8 × 10^−4^ j	4265.80	296.41	1.96 × 10^−5^ (mean) 7.96 × 10^−4^ (max)	n.a. (mean) 0.05 (LoQ)^k^	241.19
AMA	–	–	–	90^i^	1.3 × 10^−4^ l	151.36	151.25	0.0038 (mean) 0.214 (max)	n.a. (mean) 0.01 (LoQ)^k^	234.42
CBZ	0.0467 (children) 0.0675 (adults; average)^h^,^m^,^n^	–	–	100^i^	5.5 × 10^−5^ o	251.19	236.27	0.0414 (mean) 1.47 (max)	n.a. (mean) 0.01 (LoQ)^k^	22.39
CIP	0.0021^p^	–	–	69^q^	1.7 × 10^−6^ l	12.59	331.35	0.0049 (mean) 0.434 (max)	n.a. (mean) 0.05 (LoQ)^k^	147.91
CYC	0.0001639^l^	0.61^r^	0.0001^s^	97^t^,^u^	5.7 × 10^−6^ l	16.98	261.09	2.07 × 10^−4^ (mean) 0.00948 (max)	n.a. (mean) 0.01 (LoQ)^k^	3.24
DCF	0.0042^p^	–	–	97^i^	1.9 × 10^−4^ j	4570.88	296.15	0.0219 (mean) 1.81 (max)	n.a. (mean) 0.3 (LoQ)^k^	275.42
DOX	0.00003^p^	–	–	85^t^,^u^	3.4 × 10^−8^ l	0.46	444.44	0.00837 (mean) 0.313 (max)	n.a. (mean) 0.05 (LoQ)^k^	58.88
ERY	0.013^p^	–	–	35^i^	2.6 × 10^−8^ l	97.72	733.94	0.0186 (mean) 2.03 (max)	n.a. (mean) 0.01 (LoQ)^k^	69.18
IOP	–	–	–	60^t^,^u^	4.1 × 10^−10^ l	0.68	777.09	0.00678 (mean) 0.155 (max)	0.008 (mean) 0.013 (max)	3.16
MET	0.0318 (average)^p^,^v^	–	–	54^i^	1.4 × 10^−6^ l	0.18	129.17	0.0845 (mean) 3.14 (max)	n.a. (mean) 0.05 (LoQ)^k^	1.35
MEP	0.0075 (average)^p^,^w^	–	–	96^i^	2.5 × 10^−5^ x	151.36	267.37	0.0369 (mean) 1.47 (max)	n.a. (mean) 0.01 (LoQ)^k^	8.13
OXA	–	–	–	97^i^	2.2 × 10^−5^ l	190.55	286.72	0.0142 (mean) 0.44 (max)	n.a. (mean) 0.05 (LoQ)^k^	72.44
PHE	0.036^w^	–	–	98^i^	2.8 × 10^−5^ l	37.15	188.23	2.09 × 10^−5^ (mean) 2.62 × 10^−3^ (max)	n.a. (mean) 0.01 (LoQ)^k^	9.33
SUL	–	–	–	95^i^	3.0 × 10^−6^ l	9.77	278.34	n.a. (mean)^y^ n.a. (max)^y^	n.a. (mean) 0.05 (LoQ)^k^	22.91
VAL	0.0033^p^	–	–	55^i^	2.5 × 10^−5^ l	4265.80	435.53	0.028 (mean) 1.15 (max)	n.a. (mean) 0.01 (LoQ)^k^	6.92

*Note*: For details on the data input selection and associated assumptions, see the Supporting Information.

^a^ Daina et al. (2017).

^b^ Kim et al. (2021).

^c^ Duarte et al. (2022).

^d^ Lämmchen et al. (2021).

^e^ Gunnar G. Niebaum, USF, Osnabrück University (personal communication, 6^th^ November 2020).

^f^ Waterbedrijf Vitens (personal communication, 1^st^ June 2021); according to Vitens, drinking water supplied complies with Dutch legal water quality requirements.

^g^ Benfenati et al. (2013).

^h^ Kumar and Xagoraraki (2010).

^i^ Shen et al. (2010).

^j^ Chen et al. (2007).

^k^ Maximum concentrations assumed to be equal to the pharmaceuticals' highest limit of analytical quantification (LoQ). For pharmaceuticals for which no chemical analysis data were available in drinking water (i.e., 17α‐ethinylestradiol, metformin, oxazepam), an assumed LoQ of 0.05 µg/L was applied, n.a., substances for which measurement information was not available.

^l^ This study.

^m^ Bull et al. (2014).

^n^ Cunningham et al. (2010).

^o^ Fourie et al. (2004).

^p^ Suchomel et al. (2015).

^q^ Palm et al. (1997).

^r^ Cal/EPA (1992).

^s^ NL (2012).

^t^ Cheng et al. (2012).

^u^ Hou et al. (2007).

^v^ Schwab et al. (2005).

^w^ Schriks et al. (2010).

^x^ Modamio et al. (2000).

^y^ The GREAT‐ER model used in respective data sources does not account for veterinary emission sources; therefore, concentration predictions for sulfamethazine, which is most exclusively used in veterinary medicine, were not available (n.a.).

### Human behavior

Human behavior determines the extent to which people are in contact with polluted water, either directly or indirectly, that is, via recreational swimming, drinking water, and fish consumption. We defined five archetypes of human behavior:

(A) The “Average” archetype refers to adult individuals whose behavior falls within the typical range of expectable behavior in the majority of the population;
(F) The “Fisherman” archetype refers to adult individuals with high consumption of fish caught in the Vecht River;
(S) The “Swimmer” archetype refers to adult individuals who heavily engage in frequent swimming activities in the Vecht River;
(D) The “Drinker” archetype refers to adult individuals who differ from the “average” archetype in their unusual high consumption of Vecht‐derived drinking water; and
(E) The “Extreme” archetype refers to adult individuals with combined characteristics of the “Fisherman,” “Swimmer,” and “Drinker” archetypes.

The lifetime‐averaged daily pharmaceutical uptake of all archetypes was calculated assuming typical behavior at nonadult life stages. Human physical and behavioral data were mostly informed by the Dutch population characteristics; it was assumed that the German population characteristics resemble these.

### Exposure scenarios

An exposure scenario combines an assumption about the API concentrations present in surface and drinking water (I, II, or III) with a distinct type of human behavior (A, F, S, D, or E). In total, we calculated exposure and risk for 15 scenarios, that is, three environmental exposure levels for each of the five human archetypes. Table 3 presents the pharmaceutical‐specific input parameters used in the exposure model calculations, and in Table 4, the age‐ and behavior‐specific input parameters are presented.

**Table 4:** Age‐ and behavior‐specific input values for lifetime uptake and hazard estimation

Age group	$q_{s}$ (mL/min)^a^	$t_{e}$ (min/event)^a^	$s_{e}$ (events/year)^a^	$q_{w}$ (mL/day)^b^	$q_{f}$ (mg/day)^b^	$f_{\mathrm{HSA}}$ (%)^c^	$S_{f}$ a	h(cm)	m(kg)
0–1	0.0	0	0.0	350	0.0	19	59	65.7^d^	7.2^d^
1–5	0.5	79	8.0	425	52 600	3	59	91.7^d^	13.7^d^
5–10	0.5	79	8.0	583	69 960	3	59	125.6^d^	25.0^d^
10–18	0.4	67.9	7.6	951	67 750	3	54	161.7^d^	49.6^d^
18–80	0.4^A, F, D^ 0.58 (95^th^)^S, E^	54^A, F, D^ 151.8 (95^th^)^S, E^	7.0^A, F, D^ 18.8 (95^th^)^S, E^	1757^A, F, S^ 4218 (95^th^)^D, E^	108 969^A, S, D^ 278 002 (95^th^)^F, E^	3	45	174.2^e^	78.4^e^

*Note*: For details on the data input selection and associated assumptions, see the Supporting Information.

Abbreviations: 95^th^, ninety‐fifth percentile; A, “Average” behavior archetype; D, “Drinker” behavior archetype; E, “Extreme” behavior archetype; F, “Fisherman” behavior archetype; S, “Swimmer” behavior archetype.

^a^Schets et al. (2011).

^b^van Rossum, et al. (2020).

^c^Livingston and Lee (2000).

^d^Fredriks et al. (2000).

^e^CBS (2019).

### Combined effects and risks of APIs

Pharmaceutical mixture risks were estimated by summing individual HQ, implicitly assuming that the APIs have a similar mode of action, but do not affect each other's toxicity (noninteractive), that is, the (concentration) addition‐based hazard index (*HI*_add_). However, actual combined effects of APIs could be more than additive (synergism, potentiation) or less than additive (antagonism, inhibition, masking) (More et al., 2019). To accommodate this, pairwise drug interaction information was incorporated into the estimation of risk indices, following the concept of an interaction‐based hazard index (*HI*_int_, Table 2—Equation 15) (USEPA, 2000, 2007). Interaction information for each pharmaceutical pair in the mixture was expressed by an interaction magnitude (*M*) and a weight‐of‐evidence (*B*) factor. Factor *M* represents the mutual influence of the pair on their combined toxicity. Values of *M* were obtained from Roden et al. (2015) and USFDA (2012), in line with the type of interaction severity reported by the Drugbank Interaction Checker^©^ (Wishart et al., 2018). In the present study, all interactions were identified as one‐way interactions, that is, the interaction effect is exerted by one of the components. Factor *B* represents the quality of the data and the direction of the drug interactions. The direction of API pairwise effects is determined by the sign of *B*, ranging from −1 for less than additive interactions, to +1 for more than additive interactions. In a mixture with no pairwise interactions (*B*_*ij*_ = 0), the additivity assumption prevails (*HI*_int_ = *HI*_add_). In the present study, interaction directions were conservatively assumed to be |*B*| = 1. Hazard index equations are presented in Table 2, and input parameters are detailed in Tables S11–S13.

## RESULTS

**Figure 3:**
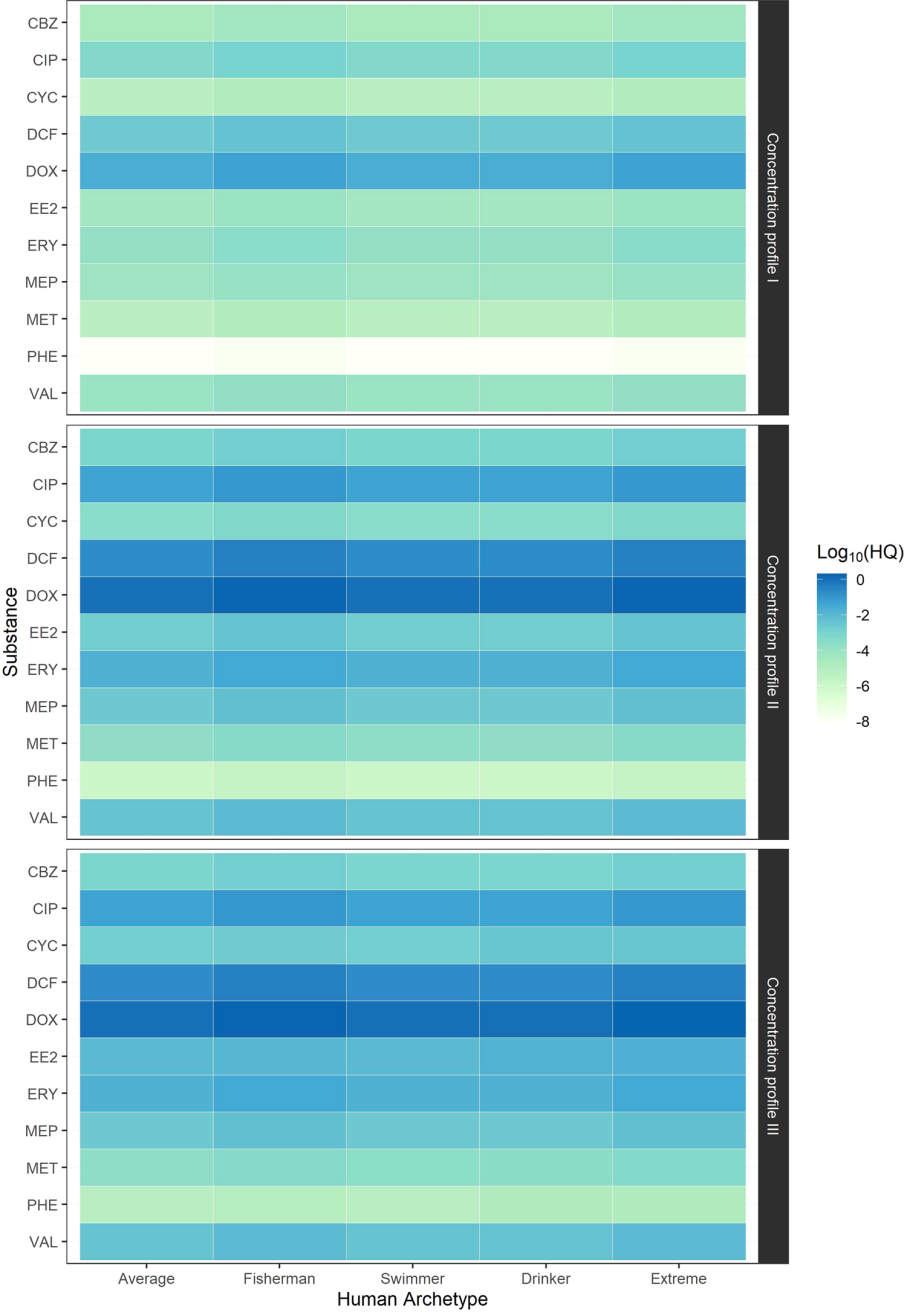
Human health lifetime hazard quotients (HQ) of studied pharmaceuticals in the Vecht River catchment. CBZ, carbamazepine; CIP, ciprofloxacin; CYC, cyclophosphamide; DCF, diclofenac; DOX, doxycycline; EE2, 17α‐ethinylestradiol; ERY, erythromycin; MEP, metoprolol; MET, metformin; PHE, phenazone; VAL, valsartan

We generated 4500 age‐ and route‐specific pharmaceutical daily uptake values for 15 APIs covering a variety of exposure conditions (Supporting Information). Aggregation of these age‐ and route‐specific uptake values resulted in 165 lifetime risk estimates for 11 APIs (Supporting Information). The daily uptake of APIs per age group is shown in Table 5. The risks calculated for the 11 remaining APIs are shown in Figure 3. The combined mixture risks of these 11 APIs, calculated following the principles of USEPA's addition‐ and interaction‐based hazard index, are listed in Table 6.

**Table 5:** Geometric mean of pharmaceutical daily uptake per age group and concentration profile for an average behavior archetype

	Pharmaceutical daily uptake (mg/kg/day)		
Age group (years)	I	II	III
0–1	0 (0%)	0 (0%)	8.86 × 10^−7^ (25%)
1–5	9.03 × 10^−10^ (16%)	4.71 × 10^−8^ (16%)	8.78 × 10^−8^ (10%)
5–10	6.37 × 10^−10^ (14%)	3.32 × 10^−8^ (14%)	6.28 × 10^−8^ (9%)
10–18	3.19 × 10^−10^ (12%)	1.66 × 10^−8^ (12%)	3.56 × 10^−8^ (8%)
18–80	2.05 × 10^−10^ (58%)	1.07 × 10^−8^ (58%)	2.66 × 10^−8^ (47%)

*Note*: The uptake values represent the aggregated daily uptake of all pharmaceuticals and exposure routes. The total pharmaceutical uptake per age group is presented as a lifetime percentage.

**Table 6:** Pharmaceutical mixture hazard indices

Concentration profile	Hazard	Average	Fisherman	Swimmer	Drinker	Extreme
I	*HQ*_max_	2.59 × 10^−2^	5.34 × 10^−2^	2.59 × 10^−2^	2.59 × 10^−2^	5.34 × 10^−2^
	*HI*_add_	2.92 × 10^−2^	6.00 × 10^−2^	2.92 × 10^−2^	2.92 × 10^−2^	6.01 × 10^−2^
	*HI*_int_	2.98 × 10^−2^	6.14 × 10^−2^	2.98 × 10^−2^	2.98 × 10^−2^	6.14 × 10^−2^
	*d*_*HI*_	2%	2%	2%	2%	2%
II	*HQ*_max_	0.97	2.00	0.97	0.97	2.00
	*HI*_add_	1.23	2.54	1.23	1.23	2.54
	*HI*_int_	1.28	2.62	1.28	1.28	2.62
	*d*_*HI*_	4%	4%	4%	4%	4%
III	*HQ*_max_	1.01	2.03	1.01	1.05	2.07
	*HI*_add_	1.28	2.58	1.28	1.33	2.64
	*HI*_int_	1.32	2.67	1.33	1.38	2.72
	*d*_*HI*_	3%	3%	3%	3%	3%

Abbreviations: *d*_*HI*_, relative change in hazard index; *HI*_add_, addition‐based hazard index; *HI*_int_, interaction‐based hazard index; *HQ*_max_, highest *HQ* in mixture (i.e., doxycycline).

The HQ for individual APIs ranged from 10^−9^ to 2.5 (Figure 3). The antibiotic doxycycline consistently had the highest calculated HQ for all exposure scenarios. The commonly used over‐the‐counter drug diclofenac showed the second highest HQ. The antibacterials ciprofloxacin and erythromycin were recurrently the third highest HQ  (10^−4^ to 10^−1^). The fourth highest risk across exposure scenarios was consistently calculated for the antihypertensive agents valsartan and metoprolol. The HQ estimated in concentration profile II, in comparison with average concentration profile I, underwent changes ranging from 34× higher for carbamazepine to 124× higher for phenazone. For the majority of pharmaceuticals, however (7 out of 11), this change was less than 45×. HQ estimated for the extreme concentration profile III, in comparison with concentration profile I, increased from 35× higher for carbamazepine to 10^3^× higher for phenazone. With the exception of doxycycline, none of the APIs evaluated in this study had an HQ exceeding the risk threshold (*HQ* = 1), not even under extreme exposure conditions, implying that the predicted lifetime exposure did not exceed health safety thresholds.

An individual's average daily uptake of APIs showed age dependency (Table 5). Young age groups were systematically associated with higher uptake values per kilogram body weight. Under average environmental conditions (I), toddlers and adults contribute 16% and 58% to the total lifetime uptake, respectively. Under extreme environmental exposure conditions (III), infants and adults contribute 25% and 47% to the total lifetime uptake, respectively.

The HI associated with combined exposure to pharmaceutical mixtures showed a wide range across the simulated exposure scenarios (Table 6). Pharmaceutical mixture risks ranged from 10^−2^ to 2.6 when assuming additive biological effects (*HI*_add_), and from 10^−2^ to 2.7 when accounting for biological interactions (*HI*_int_). The lowest *HI*_add_ and *HI*_int_ were associated with the “Average,” “Swimmer,” and “Drinker” archetypes under concentration profile I, whereas the highest were associated with the “extreme” archetype under concentration profile III. The average differences between *HI*_add_ and *HI*_int_ in concentration profiles I, II, and III were 2%, 4%, and 3%, respectively.

## DISCUSSION

Three main observations stand out from the results (Figure 3). First, scenarios of high exposure resulted in the highest risks, unsurprisingly so due to assuming maximum surface and drinking water concentrations. Second, fish consumption was the exposure route that contributed most to elevated risks. Third, drug interactions only marginally increase health risks due to simultaneous pharmaceutical exposure (up to a 4% increase of *HI*_add_). These observations emphasize that health risks are strongly dictated by pharmaceutical environmental concentrations, followed by human behavioral differences.

The high HQ for doxycycline, the only API exceeding its ISD, is the result of a relatively low ISD (0.03 µg/kg/day). For this particular API, subjective choices and interpretations (e.g., relating to the uncertainty factors applied) are known to substantially influence the ISD, resulting in differences up to three orders of magnitude (Kumar et al., 2010). Here, we used the lowest ISD reported in the public literature, resulting in an *HQ* of 2.1 for the most extreme scenario (E‐III). Choosing a higher ISD would have resulted in acceptable lifetime risks (*HQ* < 1), even under extreme exposure conditions. Estimated safe reference levels can vary widely depending on the derivation procedure, selection of population and health endpoints, and their perceived uncertainty. This ambiguity illustrates the impact of ISDs in estimated risks. The practical implication is that, next to exposure reduction measures, reducing the uncertainty in acceptable exposure levels can improve the scientific underpinning for estimating risks, often reducing the need to apply a conservative bias to avoid underestimating risks.

Diclofenac had the second highest HQ (up to 0.4). The concentration of diclofenac in surface and drinking water was comparable to the other APIs (Table 3); yet, its lifetime uptake estimates were substantially higher. Diclofenac uptake was estimated to occur via the skin during swimming. However, for individuals consuming contaminated fish, eating becomes the dominant route of exposure (~100%). These observations are in line with diclofenac's properties, that is, its very high skin permeability coefficient (0.19 mm/min), its relatively high octanol–water partition coefficient, its low molecular weight, and its ability to accumulate in fish lipid tissue. Diclofenac's estimated bioconcentration factor was 0.275 ml/mg, being in close agreement with experimental values (Cuklev et al., 2011).

In most exposure scenarios, pharmaceutical uptake mainly occurred via fish consumption, followed, to a small extent, by surface water ingestion and dermal absorption during swimming activities. Generally, pharmaceuticals with relatively high hydrophilicity were taken up after accidental swallowing of water during swimming events (e.g., iopamidol, doxycycline, erythromycin, ciprofloxacin, metformin), whereas pharmaceuticals with relatively high hydrophobicity were taken up via dermal absorption (e.g., 17α‐ethinylestradiol, amantadine, diclofenac).

The risks posed by pharmaceutical mixtures were estimated to be higher than any individual pharmaceutical (Table 6). Still, the increased risk was limited, even assuming relatively conservative (i.e., high‐end exposure) exposures due to the low percentage of major drug interaction effects (<7%; Table S14). Estimated HIs did not surpass ISDs (*HI* < 1) under average pharmaceutical concentrations (I). This suggests that lifetime health risks due to direct toxicity associated with the intake of the 15 selected APIs from the Vecht River would not be expected. However, should additional APIs be assessed, risk estimates are likely to become higher.

Despite the improbable occurrence of exceptional exposure conditions (e.g., concentration profile III in combination with the “Fisherman” and “Extreme” behavior archetypes), these scenarios aid the identification of key exposure factors, including risky behaviors. The highest lifetime risks were found to be associated with the “Fisherman” and “Extreme” behavior archetypes, where the latter inherited the risks of the former, indicating that pharmaceutical uptake via fish consumption could be an important exposure route for these individuals (Kumar & Xagoraraki, 2010). However, it should be noted that the present study conservatively assumes that all consumed fish are sourced from the Vecht River. Consumption of fish from other origins will result in different risk estimates, likely to be much lower than those reported for concentration profile III (Bean et al., 2018; Rojo et al., 2019). Xie et al. (2019) reported that health risks associated with pharmaceutical contaminated fish are negligible, although factors like dietary habits were not accounted for.

When API concentrations in drinking water were assumed to equal the limit of quantification (LoQ) (concentration profile III), the lifetime risk for “Drinker” archetypes increased by 92%, 91%, and 88% for phenazone, cyclophosphamide, and 17α‐ethinylestradiol, respectively. This indicates the potential importance of drinking water as a relevant exposure route (Santos et al., 2020), although the absolute risks were still low, which is supported by other studies (Houtman et al., 2014). These results also emphasize the importance of increasing the reliability of analytical quantification, given that the assumption that drinking water concentrations matched the LoQ greatly affected the risk estimates associated with drinking water.

Human metabolites and environmental TPs of APIs are often found in the aquatic environment (Ma et al., 2020). The ecotoxicological effects, environmental fate, and risk of these metabolites and TPs are increasingly being studied and assessed (Maculewicz et al., 2022; Wang et al., 2021). The present exposure model allows the inclusion of these compounds, provided that the necessary parameter adjustments are made. However, adverse effect levels for metabolites and TPs in humans, and a detailed profiling of these substances in the Vecht River are missing. We therefore did not include metabolites and TPs in our assessment. This effectively means that we likely underestimate the true human risk, particularly for APIs that are extensively metabolized or transformed, and if these metabolites and TPs are toxic to humans (de Jongh et al., 2012; Zind et al., 2021). Despite uptake during childhood contributing less to lifetime uptake than uptake during adulthood, it represents almost half of an individual's total lifetime uptake (Table 5). This can be explained by the high body surface to body weight ratio and high energy demand resulting in a high contaminant uptake per body mass unit (Ferguson et al., 2017; OECD, 2019). These observations point to the potential relevance of understanding age‐specific susceptibilities of long‐term exposure to low levels of APIs such as differences in gastrointestinal absorption, skin characteristics, and renal and liver functions (Bruckner, 2000). Analysis of other population groupings could also be of interest and reveal sensitive subpopulations, such as pregnant and lactating women (Beszterda & Frański, 2018).

**Figure 4:**
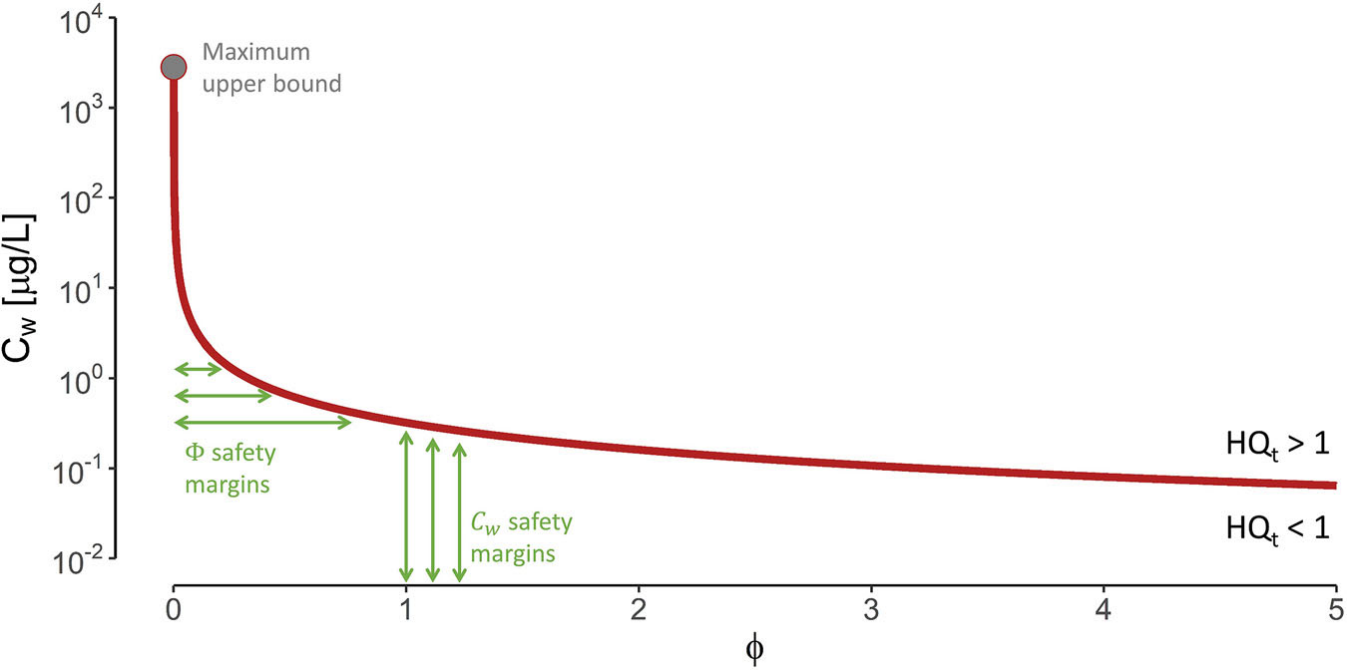
Surface water concentrations compared to hazard quotient (HQ) for doxycycline (*C*_w_) in relation to the fish consumption of the target population (Φ). The red curve depicts the target hazard quotient of 1 (*HQ*_t_ = 1)

Risk quantification is typically the result of a reactive approach, from which an exposure‐based HQ is estimated. However, HQ can be repurposed as a target risk value (*HQ*_t_) in a proactive approach, from which protective exposure limits are derived. The latter can be of particular interest to water managers in search of pragmatic tools for risk prevention, mitigation, or reduction. Thus, our exposure model can be rearranged in light of risk acceptance criteria. To illustrate this, we derived an exemplary equation on the relationship between pharmaceutical concentration in surface water (*C*_w_) and fish consumption of a target population (Φ). For details on the equation's derivation, see the Supporting Information. The maximum acceptable pharmaceutical concentration in surface water can be estimated once the amount of its fish consumed by the target population is established, or vice versa (Figure 4). An increase in fish consumption leads to a rapid decrease in the permissible concentration of the pharmaceutical in surface water. For example, to prevent exceedance of the target risk benchmark (*HQ*_t_ = 1) in a population consuming twice as much fish as the national average (Φ = 2), diclofenac and doxycycline water concentrations should be kept below 5 and 0.2 µg/L, respectively. In other words, Vecht River water concentrations of diclofenac and doxycycline can be 228 and 24× higher than present average concentrations before the risk is deemed unacceptable. Due to remaining pharmaceutical exposure from swimming and drinking water, even in the absence of fish consumption (Φ = 0), concentration limits for diclofenac and doxycycline are 58 and 3 mg/L, respectively. Conversely, the lifetime consumption of fish from the Vecht River with average diclofenac and doxycycline water concentrations would have to be 441 and 40× higher than the national average consumption to meet the risk threshold (*HQ* = 1). By using these versatile guides, water managers can readily gain insight into the potential human health risks based on minimal information, bypassing unnecessary and laborious risk assessment.

## CONCLUSION

Human health risks from direct toxicity associated with the lifetime exposure to pharmaceutical residues in the Vecht's River catchment were largely less than safe limits. Most individuals in contact with Vecht River water are far from exceeding acceptable risk levels (10^−2^ < *HQ* < 10^−9^). Exceptionally, only in high water contamination conditions such as river segments immediately downstream a wastewater treatment plant's (WWTP) effluent emission point did exposure to the antibiotic doxycycline pose an appreciable risk (*HQ* < 2) to individuals who daily consumed 229 g of contaminated fish caught at those locations. The cumulative risk of pharmaceutical mixtures also did not exceed safe limits under normal conditions. However, long‐term daily exposure to highly contaminated sites in the Vecht River is discouraged due to the potential health risks (1.3 < *RI* < 2.6), particularly via fish consumption. European regulatory authorities have not issued specific fish consumption advisories for APIs, but the EU is currently considering including selected APIs on the priority substances list. If this becomes reality, water quality standards will be derived covering exposure through fish consumption. From a global perspective, pharmaceutical residue concentrations in other world regions have been found to be 10 to 10^4^ higher than in the current study (Eike et al., 2019), indicating likely higher health risks at those locations.

We show that key human features and activities, and environmental parameters of varied complexity can be integrated into a relatively simple deterministic exposure model to estimate lifetime health risks of pharmaceuticals in the water environment. The exposure model presented is also applicable to metabolites and TPs, provided that adjustments are made. The utility of the exposure model still relies on data quality and availability, namely, data about the end use of the surface water body of interest. A valuable first step would be for water managers to comprehensively survey the types of water usage at relevant sites. Once the most relevant water‐related activities are identified and their associated risks are assessed, risk management strategies can then be customized to specific locations, to more efficiently restrict health risks. For example, substance prioritization and monitoring could be informed based on a substance's bioaccumulation, persistence, or permeability for surface waters often used for fishing, drinking water production, or swimming, respectively.

When prioritizing resources to estimate human health risks, we recommend that water managers collect basic information on (1) the consumption of fish from sites downstream of WWTP facilities, and (2) the consumption and environmental releases of diclofenac, doxycycline, or compounds with similar permeability and bioaccumulation potential. With increased availability of empirical site‐specific information, the screening approach can be turned into a site‐specific assessment, improving the accuracy of the risk estimates.

Ultimately, the present study renders laborious risk assessments unnecessary by proposing a simple method to pragmatically determine whether health standards for APIs are likely to be exceeded based on local environmental conditions and population behavior.

## CONFLICT OF INTEREST

The authors declare no conflict of interest.

## AUTHOR CONTRIBUTIONS

**Daniel J. Duarte**: conceptualization, methodology, formal analysis, investigation, writing—original draft, writing—review & editing, visualization; **Rik Oldenkamp**: conceptualization, writing—review & editing, supervision; **Ad M. J. Ragas**: conceptualization, writing—review & editing, supervision, project administration, funding acquisition.

## Supporting information

This article contains online‐only Supporting Information.

Supporting Information_I.docx contains a comprehensive collection of data used in the human exposure model.Supporting Information_II.docx contains the results of the simulations using the human exposure model. Supporting Information_III.docx contains the derivation of an exemplary equation on the relation between exposure factors based on target hazard quotients. Supporting Information_IV.docx contains the graphical abstract.

## Data Availability

Data can be provided upon request by the authors. For data requests, please contact Daniel J. Duarte (daniel.duarte@ru.nl) or Ad M. J. Ragas (ad.ragas@ru.nl).
