# Supplementary material for: Human health risk assessment of pharmaceuticals in the European Vecht River
Source: Integr Environ Assess Manag. 2022 Feb 28;18(6):1639–54. doi: 10.1002/ieam.4588 (PMC9790459; doi:10.1002/ieam.4588)
Supplement: Supplementary file 1 — Supporting Information_I.docx contains a comprehensive collection of data used in the human exposure model.Supporting Information_II.docx contains the results of the simulations using the human exposure model. Supporting Information_III.docx contains the derivation of an exemplary equation on the relation between exposure factors based on target hazard quotients. Supporting Information_IV.docx contains the graphical abstract. [file IEAM-18-1639-s002.docx]

**SUPPORTING INFORMATION**

**TITLE**

Human Health Risk Assessment of Pharmaceuticals in the European Vecht River

**AUTHORS**

Daniel J. Duarte ^a^, Rik Oldenkamp ^b^, Ad M. J. Ragas ^a, c^

*^a^ Radboud University Nijmegen, Institute for Water & Wetland Research, Department of Environmental Science, PO Box 9010, NL-6500 GL, Nijmegen, The Netherlands*

*^b^ Department of Global Health-Amsterdam Institute for Global Health and Development, Amsterdam UMC, University of Amsterdam, Paasheuvelweg 25, NL-1105 BP, Amsterdam, The Netherlands*

*^c^ Open University, Faculty of Science, Department of Environmental Sciences,* *Valkenburgerweg 177, 6419 AT, Heerlen, The Netherlands*

**TABLE OF CONTENTS**

[**TABLE S1.** Average body height, weight and surface area (*A_s_*) of the Dutch population. 3](#_Toc78912203)

[**TABLE S2.** Swimming exposure parameters of fresh water swimmers in The Netherlands. 4](#_Toc78912204)

[**TABLE S3.** Probability of full body submergence ($S_{f})$, fraction of head surface ($f_{\mathrm{HSA}})$and total fraction of exposed skin ($f_{s})$ in a swimming event. 5](#_Toc78912205)

[**TABLE S4.** Drinking water consumption in The Netherlands. 6](#_Toc78912206)

[**TABLE S5.** Fish consumption in The Netherlands. 7](#_Toc78912207)

[**TABLE S6.** Octanol-water partition coefficients, molecular weights and skin permeability coefficients of pharmaceutical active ingredients (API). 8](#_Toc78912208)

[**TABLE S7.** Steady-state bioconcentration factor (BCF) estimates. 9](#_Toc78912209)

[**TABLE S8.** Annual concentrations of substances measured in drinking water from Viten’s Vechterweerd treatment plant in Zwolle. 10](#_Toc78912210)

[**TABLE S9.** Human gastrointestinal absorption fractions (*f_GI_*). 11](#_Toc78912211)

[**TABLE S10.** Health-based reference doses. 12](#_Toc78912212)

[**TABLE S11.** Severity of drug interactions (*M*) of active pharmaceutical ingredient (API) pairs. 13](#_Toc78912213)

[**TABLE S12.** Magnitude of drug interactions (*M*) of active pharmaceutical ingredient (API) pairs. 14](#_Toc78912214)

[**TABLE S13.** Binary weight-of-evidence factor (*B*) of drug interactions between pairs of active pharmaceutical ingredient (API). 15](#_Toc78912215)

[**TABLE S14.** Categorisation of drug interaction effects for all 210 pairs of active pharmaceutical ingredients in the present study. 16](#_Toc78912216)

# **TABLE S1.** Average body height, weight and surface area (*A_s_*) of the Dutch population.

| Age  [year] ^a^ | Height  [cm] | Weight  [kg] | A_s_  [cm^2^] ^d^ |
| --- | --- | --- | --- |
| 0-1 ^b^ | 65.7 | 7.2 | 3531 |
| 1-5 ^b^ | 91.7 | 13.7 | 5893 |
| 5-10 ^b^ | 125.6 | 25.0 | 9577 |
| 10-18 ^b^ | 161.7 | 49.6 | 15385 |
| 18-80 ^c^ | 174.2 | 78.4 | 19725 |
| ^a^ Weighted average was applied to the height and body weight values according to the age ranges used in this assessment (i.e. 0-1 year, 1-5 years, 5-10 years, 10-18 years). ^b^ Fredriks et al. (2000) ^c^ CBS (2019) ^d^ Estimated according to Lee et al. (2008). | | | |

# **TABLE S2.** Swimming exposure parameters of fresh water swimmers in The Netherlands.

|  | Frequency  [event/year] | | Duration  [min/event] | | Intake  [ml/min] | |
| --- | --- | --- | --- | --- | --- | --- |
| Age Group  [year] ^a, b^ | Mean | 95^th^ | Mean | 95^th^ | Mean | 95^th^ |
| 0-1 ^c^ | 0.0 | 0.0 | 0.0 | 0.0 | 0.0 | 0.0 |
| 1-5 | 8.0 | 22.4 | 79.0 | 225.0 | 0.5 | 0.58 |
| 5-10 | 8.0 | 22.4 | 79.0 | 225.0 | 0.5 | 0.58 |
| 10-18 | 7.6 | 20.8 | 67.9 | 192.4 | 0.4 | 0.58 |
| 18-80 | 7.0 | 18.8 | 54.0 | 151.8 | 0.4 | 0.58 |
| Data based on Schets et al. (2011) ^a^ Male and female values were averaged. ^b^ Schets et al. (2011) differentiate between childhood and adulthood, but do not specify the corresponding age ranges. We applied the childhood values for the 1-18 year age class and the adulthood values for the 18-80 year age class. ^c^ Children up to 1 year of age were assumed not to engage in swimming activities in freshwater. Frequency = number of swimming events per year. Duration = activity time per swimming event. Volume = quantity of swimming water swallowed during a swimming event. Intake = quantity of swimming water swallowed per minute of swimming event. 95th = ninety fifth percentile | | | | | | |

# **TABLE S3.** Probability of full body submergence ($S_{f})$, fraction of head surface ($f_{HSA})$and total fraction of exposed skin ($f_{s})$ in a swimming event.

| Age group  [year] | $S_{f}$ ^a^ | $f_{HSA}$ ^b^ | $f_{s}$ |
| --- | --- | --- | --- |
| 0-1 | 0.59 | 0.19 | 0.92 |
| 1-5 | 0.59 | 0.03 | 0.99 |
| 5-10 | 0.59 | 0.03 | 0.99 |
| 10-18 | 0.54 | 0.03 | 0.99 |
| 18-80 | 0.45 | 0.03 | 0.98 |
| ^a^ Schets et al. (2011), ^b^ Livingston and Lee (2000) | | | |

# **TABLE S4.** Drinking water consumption in The Netherlands.

| Ages  [year] | Mean  [ml/day] | 5^th^  [ml/day] | 50^th^  [ml/day] | 95^th^  [ml/day] |
| --- | --- | --- | --- | --- |
| 0-1 ^a^ | 350 | 0 | 343 | 671 |
| 1-5 ^b^ | 425 | 51 | 338 | 1039 |
| 5-10 ^b^ | 583 | 108 | 440 | 1525 |
| 10-18 ^b^ | 951 | 169 | 709 | 2508 |
| 18-80 ^b^ | 1757 | 299 | 1448 | 4218 |
| ^a^ Data on recommended infant milk formula intake obtained from the Youth Health Care Utrecht (https://www.jeugdengezinutrecht.nl). Data on intake frequency of infant milk formula obtained from Engelse and van Dommelen, (2020). Data on drinking water fraction of milk formula preparations (i.e. 90%) obtained from HiPP (https://www.hipp.nl). ^b^ Data obtained from the Dutch National Consumption Survey 2012-2016 (RIVM 2018). Water intake refers to drinking water and other water-containing beverages such as coffee and tea. Consumption values were weight averaged according to the associated age ranges. 5^th^ = fifth percentile. 50^th^ = fiftieth percentile. 95^th^ = ninety fifth percentile. | | | | |

# **TABLE S5.** Fish consumption in The Netherlands.

| Ages  [year] | Mean  [mg/day] | 5^th^  [mg/day] | 50^th^  [mg/day] | 95^th^  [mg/day] |
| --- | --- | --- | --- | --- |
| 0-1 ^a^ | 0 | 0 | 0 | 0 |
| 1-5 | 52600 | 8625 | 40150 | 133500 |
| 5-10 | 69960 | 9940 | 66480 | 160800 |
| 10-18 | 67750 | 10450 | 50000 | 168050 |
| 18-80 | 108969 | 12610 | 94077 | 278002 |
| Data obtained from the Dutch National Consumption Survey 2012-2016 (RIVM 2018). Consumption values were weight averaged according to the associated age ranges. Fish soft tissue was assumed in the present study to be consumed in its entirety. 5^th^ = fifth percentile. 50^th^ = fiftieth percentile. 95^th^ = ninety fifth percentile. ^a^ Fish consumption in infants assumed to be zero because solid foods are introduced gradually to infants only after 6 months and Dutch culture strongly favours fruits and vegetables. | | | | |

# **TABLE S6.** Octanol-water partition coefficients, molecular weights and skin permeability coefficients of pharmaceutical active ingredients (API).

| API | log K_ow_ ^a^ | MW  [g/mol] | log k_p_  [cm/sec] ^i^ | k_p_  [cm/sec] | k_p_  [cm/min] |
| --- | --- | --- | --- | --- | --- |
| 17α-Ethinylestradiol | 3.63 | 296.41 | -5.53 ^b. d. e^ | 2.9·10^-6^ | 1.8·10^-4^ |
| Amantadine | 2.18 | 151.25 | -5.67 ^c^ | 2.1·10^-6^ | 1.3·10^-4^ |
| Carbamazepine | 2.40 | 236.27 | -6.04 ^b. g^ | 9.2·10^-7^ | 5.5·10^-5^ |
| Ciprofloxacin | 1.10 | 331.35 | -7.54 ^c^ | 2.9·10^-8^ | 1.7·10^-6^ |
| Cyclophosphamide | 1.23 | 261.09 | -7.02 ^c^ | 9.6·10^-8^ | 5.7·10^-6^ |
| Diclofenac | 3.66 | 296.15 | -5.51 ^b. e^ | 3.1·10^-6^ | 1.9·10^-4^ |
| Doxycycline | -0.34 | 444.44 | -9.25 ^c^ | 5.6·10^-10^ | 3.4·10^-8^ |
| Erythromycin | 1.99 | 733.94 | -9.36 ^c^ | 4.3·10^-10^ | 2.6·10^-8^ |
| Iopamidol | -0.17 | 777.09 | -11.16 ^c^ | 6.9·10^-12^ | 4.1·10^-10^ |
| Metformin | -0.75 | 129.17 | -7.62 ^c^ | 2.4·10^-8^ | 1.4·10^-6^ |
| Metoprolol | 2.18 | 267.37 | -6.38 ^b. h^ | 4.1·10^-7^ | 2.5·10^-5^ |
| Oxazepam | 2.28 | 286.72 | -6.43 ^c^ | 3.7·10^-7^ | 2.2·10^-5^ |
| Phenazone | 1.57 | 188.23 | -6.33 ^c^ | 4.6·10^-7^ | 2.8·10^-5^ |
| Sulfamethazine | 0.99 | 278.34 | -7.29 ^c^ | 5.1·10^-8^ | 3.0·10^-6^ |
| Valsartan | 3.63 | 435.53 | -6.38 ^c^ | 4.2·10^-7^ | 2.5·10^-5^ |
| ^a^ Log K_ow_ values are average estimates of five predictive models (iLOGP, XLOGP3, WLOGP, MLOGP, SILICOS-IT) obtained from SwissADME (Daina et al. 2017), ^b^ measured value, ^c^ estimated value, ^d^ average value, ^e^ Chen et al. (2007), ^f^ Lee et al. (2010), ^g^ Fourie et al. (2004), ^h^ Modamio et al. (2000), ^i^ Skin permeability estimated according to the quantitative structure–property relationship (QSAR) model developed by Potts and Guy (1992). Like Potts and Guy (1992) model, most QSAR models ignore the ionization state of the permeant and ultimately their ability to diffuse across the skin. Pharmaceuticals are typically weak acids and/or bases which makes them ionization-prone with changing pH (Manallack 2007), emphasizing the limitations of widely used human skin permeability prediction models (Baba et al. 2017). Despite these limitations, these models still offer reasonably good predictions (R^2^ = 0.676-0.735, Error = 0.091-0.096) according to Brown et al. (2012) and Lian et al. (2008), are comprehensible to most professionals and are easy to implement. Potts and Guy (1992) model was based on experimental data encompassing an ample range of substances, structural groups, molecular weights (18 and above 750) and log K_ow_ (-3 and +6). | | | | | |

# **TABLE S7.** Steady-state bioconcentration factor (BCF) estimates.

|  | CAESAR | | Meylan ^a^ | | KNN/Read-Across | |
| --- | --- | --- | --- | --- | --- | --- |
| Pharmaceutical | log BCF  [L/kg] | Reliability | log BCF  [L/kg] | Reliability | log BCF  [L/kg] | Reliability |
| 17α-Ethinylestradiol | 2.02 | Low | 2.09 | Low | 2.4 ^b^ | Good |
| Amantadine | 1.48 | Low | 1.28 | Low | 2.37 ^b^ | Moderate |
| Carbamazepine | 1.35 ^b^ | Low | 1.28 | Low | 1.15 | Low |
| Ciprofloxacin | 0.32 | Low | 0.5 | Low | 2.17 ^b^ | Low |
| Cyclophosphamide | 0.51 ^b^ | Low | 0.5 | Low | -0.07 | Low |
| Diclofenac | 2.7 | Moderate | 0.5 | Low | 2.44 ^b^ | Good |
| Doxycycline | -0.17 | Low | 0.5 | Low | 1.77 ^b^ | Moderate |
| Erythromycin | -1.65 | Low | 1.69 | Low | 1.84 ^b^ | Low |
| Iopamidol | -0.46 | Low | 0.5 ^b^ | Low | 0.25 | Low |
| Metformin | 0.24 | Low | 0.5 | Low | 0.13 ^b^ | Moderate |
| Metoprolol | 0.33 | Moderate | 0.91 ^b^ | Moderate | n.a. | Low |
| Oxazepam | 0.83 | Low | 1.14 | Low | 1.86 ^b^ | Low |
| Phenazone | 0.77 | Low | 0.5 | Low | 0.97 ^b^ | Low |
| Sulfamethazine | 0.43 | Low | 0.5 | Low | 1.36 ^b^ | Moderate |
| Valsartan | 0.8 | Low | 0.5 | Low | 0.84 ^b^ | Low |
| Bioconcentration factors were estimated using the QSAR-based tool VEGA HUB (www.vegahub.eu), which encompasses the CAESAR, KNN/Read-Across and Meylan models ^a^ Sole model that includes ionic compounds in its applicability domain. ^b^ Predicted values classified with moderate and good reliability were selected for the present study. Preference was given to the value with the highest reliability. If all model outputs retrieved low reliable predictions, the highest BCF estimate was used. | | | | | | |

**TABLE S8.** Annual concentrations of substances measured in drinking water from Viten’s Vechterweerd treatment plant in Zwolle.

| Substance | Concentration ^a^ [µg/L] | | | | |
| --- | --- | --- | --- | --- | --- |
|  | 2017 | 2018 | 2019 | Mean | Max ^b^ |
| 17α-Ethinylestradiol | n.a. | n.a. | n.a. |  | 0.05 |
| Amantadine | <0.01 | <0.01 | <0.01 |  | 0.01 |
| Carbamazepine | <0.01 | <0.01 | <0.01 |  | 0.01 |
| Ciprofloxacin | <0.05 | <0.05 | <0.05 |  | 0.05 |
| Cyclophosphamide | <0.01 | <0.01 | <0.01 |  | 0.01 |
| Diclofenac | <0.01 | <0.01 | <0.30 |  | 0.30 ^c^ |
| Doxycycline | <0.05 | <0.05 | <0.05 |  | 0.05 |
| Erythromycin | <0.01 | <0.01 | <0.01 |  | 0.01 |
| Iopamidol | 0.013 | 0.005 | 0.006 | 0.008 | 0.013 |
| Metformin | n.a. | n.a. | n.a. |  | 0.05 |
| Metoprolol | <0.01 | <0.01 | <0.01 |  | 0.01 |
| Oxazepam | n.a. | n.a. | n.a. |  | 0.05 |
| Phenazone | <0.01 | <0.01 | <0.01 |  | 0.01 |
| Sulfamethazine | <0.05 | <0.05 | <0.05 |  | 0.05 |
| Valsartan | <0.01 | <0.01 | <0.01 |  | 0.01 |
| In the Netherlands, as in the Vecht River catchment, the majority of drinking water produced is sourced from groundwater but surface water can also be indirectly sourced. This is the case at Vechterweerd where surface water from the Vecht River undergoes bank filtration before being extracted for further advanced treatment. ^a^ Concentrations obtained from Dutch drinking water company Vitens (personal communication, 2021). According to Vitens, drinking water supplied complies with Dutch legal water quality requirements ^b^ Maximum concentrations assumed to be equal to the substances highest limit of analytical quantification (LoQ). For substances for which no chemical analysis data was available in drinking water (i.e. 17α-ethinylestradiol, metformin, oxazepam), an assumed LoQ of 0.05 µg/L was applied. ^c^ Occasionally there is a co-eluting substance present in the background chemical readings with the same target fragment-ion(s), compromising the quality of the quantification. In this case the the LoQ was raised to a level for which the true diclofenac concentration is confidently lower than the LoQ. n.a. = substances for which measurement information was not available. | | | | | |

# **TABLE S9.** Human gastrointestinal absorption fractions (*f_GI_*).

| Substance | *f_GI_* [%] |
| --- | --- |
| 17α-Ethinylestradiol | 100^a, c^ |
| Amantadine | 90^a, c^ |
| Carbamazepine | 100^a, c^ |
| Ciprofloxacin | 69^a, d^ |
| Cyclophosphamide | 97^b, e^ |
| Diclofenac | 97^a, c^ |
| Doxycycline | 85^b, e^ |
| Erythromycin | 35^a, c^ |
| Iopamidol | 60^b, e^ |
| Metformin | 54^a, c^ |
| Metoprolol | 96^a, c^ |
| Oxazepam | 97^a, c^ |
| Phenazone | 98^a, c^ |
| Sulfamethazine | 95^a, c^ |
| Valsartan | 55^a, c^ |
| The uptake of pharmaceutical residues was assumed to be solely determined by gastrointestinal absorption coefficients, without other pharmacokinetic and pharmacodynamic considerations (e.g. hepatic first-pass metabolism).^a^ Experimental, ^b^ Estimated, ^c^ Shen et al. (2010), ^d^ Palm et al. (1997), ^e^ Cheng et al. (2012) and Hou et al. (2007) | |

# **TABLE S10.** Health-based reference doses.

| Substance | Species ^d^ | Population ^c,d^ | *R_f_D_oral_*  [mg/kg/day] | *ISD*  [mg/kg/day] |
| --- | --- | --- | --- | --- |
| 17α-Ethinylestradiol | n.s. | n.s. | 0.000167 ^m^ | 0.0100 |
| Amantadine | - | - | - | - |
| Carbamazepine | human | children | 0.0467 ^a, g, h^ | 0.0465 |
|  |  | adults | 0.0675 ^a, g, h^ | 0.0672 |
| Ciprofloxacin | human | general | 0.0021 ^i^ | 0.0014 |
| Cyclophosphamide | human ^e^ | general | 0.0001639 ^f, j^ | 0.000159 |
| Diclofenac | human | general | 0.0042^i^ | 0.004 |
| Doxycycline | human | general | 0.00003^i^ | 0.00003 |
| Erythromycin | human | general | 0.013^i^ | 0.0046 |
| Iopamidol | - | - | - | - |
| Metformin | human | general | 0.0318 ^a, i, k^ | 0.017 |
| Metoprolol | human | general | 0.0075 ^a, i, l^ | 0.0072 |
| Oxazepam | - | - | - | - |
| Phenazone | human | general | 0.036 ^l^ | 0.0353 |
| Sulfamethazine | - | - | - | - |
| Valsartan | human | general | 0.0033 ^i^ | 0.0018 |
| ^a^ Average value. ^c^ Averaged values where named ‘general’ if values did not include ‘children’ references. ^d^ n.s. = not specified. ^e^ Human cancer potency derived from rat studies. ^f^ ${CSF}_{oral}$ of cyclophosphamide is 0.61 mg/kg/day. ^g^ Williams and Brooks (2012). ^h^ (Bull et al. 2014; Cunningham et al. 2010; Kumar and Xagoraraki 2010). ^i^ Suchomel et al. (2015). ^j^ Cal/EPA (1992). ^k^ Schwab et al. (2005). ^l^ Schriks et al. (2010). ^m^ Kumar et al. (2010). R_f_D_oral_ = reference oral dose. ISD = internal safe dose. | | | | |

# **TABLE S11.** Severity of drug interactions (*M*) of active pharmaceutical ingredient (API) pairs.

|  |  | Receptor | | | | | | | | | | | | | | |
| --- | --- | --- | --- | --- | --- | --- | --- | --- | --- | --- | --- | --- | --- | --- | --- | --- |
| Actor | API | EE2 | AMA | CBZ | CIP | CYC | DCF | DOX | ERY | IOP | MET | MEP | OXA | PHE | SUL | VAL |
|  | EE2 |  | None | None | None | None | Mod | None | None | None | Mod | None | None | Mod | None | None |
|  | AMA | None |  | Mod | Mod | None | None | None | Mod | None | None | None | None | None | None | None |
|  | CBZ | Mod | None |  | None | None | Maj | None | None | None | None | None | Mod | Maj | None | None |
|  | CIP | Maj | None | Mod |  | Maj | Maj | None | Mod | None | Mod | None | Min | Maj | None | None |
|  | CYC | Maj | None | Maj | None |  | Maj | None | None | None | None | None | None | Maj | None | None |
|  | DCF | None | Min | None | None | None |  | Min | Mod | None | Min | Mod | Min | Mod | None | None |
|  | DOX | Min | Min | Maj | Min | Maj | None |  | None | None | Min | Min | Min | None | None | None |
|  | ERY | Mod | None | Mod | None | Maj | None | None |  | None | None | None | None | None | None | None |
|  | IOP | None | None | None | None | None | None | None | None |  | Mod | None | None | None | None | None |
|  | MET | None | Mod | Mod | None | None | None | None | None | None |  | None | Min | None | None | None |
|  | MEP | None | Mod | Mod | Min | None | None | None | None | None | Mod |  | Min | None | None | None |
|  | OXA | None | Min | None | None | None | None | None | None | None | None | None |  | None | None | None |
|  | PHE | None | Min | None | None | None | None | Min | None | None | Min | Mod | Min |  | None | None |
|  | SUL | None | None | None | None | None | None | None | None | None | Mod | None | None | None |  | None |
|  | VAL | Mod | None | None | Min | Maj | Mod | None | Min | None | None | Min | None | Mod | None |  |
| Pairwise interaction severity was obtained from Drugbank Interaction Checker^©^ (Wishart et al. 2018). Pairwise symmetrical effects (2-way interactions) were not identified. AMA = amantadine. CBZ = carbamazepine. CIP = ciprofloxacin. CYC = cyclophosphamide. DCF = diclofenac. DOX = doxycycline. EE2 = 17α-ethinylestradiol. ERY = erythromycin. IOP = iopamidol. MEP = metoprolol. MET = metformin. OXA = oxazepam. PHE = phenazone. SUL = sulfamethazine. VAL = valsartan. None = no interaction identified. Min = minor interaction. Mod = moderate interaction. Maj = major interaction. | | | | | | | | | | | | | | | | |

# **TABLE S12.** Magnitude of drug interactions (*M*) of active pharmaceutical ingredient (API) pairs.

|  |  | Receptor | | | | | | | | | | | | | | |
| --- | --- | --- | --- | --- | --- | --- | --- | --- | --- | --- | --- | --- | --- | --- | --- | --- |
| Actor | API | EE2 | AMA | CBZ | CIP | CYC | DCF | DOX | ERY | IOP | MET | MEP | OXA | PHE | SUL | VAL |
|  | EE2 |  | 0,93 | 0,93 | 0,93 | 0,93 | 3,1 | 0,93 | 0,93 | 0,93 | 3,1 | 0,93 | 0,93 | 3,1 | 0,93 | 0,93 |
|  | AMA | 0,93 |  | 3,1 | 3,1 | 0,93 | 0,93 | 0,93 | 3,1 | 0,93 | 0,93 | 0,93 | 0,93 | 0,93 | 0,93 | 0,93 |
|  | CBZ | 3,1 | 0,93 |  | 0,93 | 0,93 | 5 | 0,93 | 0,93 | 0,93 | 0,93 | 0,93 | 3,1 | 5 | 0,93 | 0,93 |
|  | CIP | 5 | 0,93 | 3,1 |  | 5 | 5 | 0,93 | 3,1 | 0,93 | 3,1 | 0,93 | 1,51 | 5 | 0,93 | 0,93 |
|  | CYC | 5 | 0,93 | 5 | 0,93 |  | 5 | 0,93 | 0,93 | 0,93 | 0,93 | 0,93 | 0,93 | 5 | 0,93 | 0,93 |
|  | DCF | 0,93 | 1,51 | 0,93 | 0,93 | 0,93 |  | 1,51 | 3,1 | 0,93 | 1,51 | 3,1 | 1,51 | 3,1 | 0,93 | 0,93 |
|  | DOX | 1,51 | 1,51 | 5 | 1,51 | 5 | 0,93 |  | 0,93 | 0,93 | 1,51 | 1,51 | 1,51 | 0,93 | 0,93 | 0,93 |
|  | ERY | 3,1 | 0,93 | 3,1 | 0,93 | 5 | 0,93 | 0,93 |  | 0,93 | 0,93 | 0,93 | 0,93 | 0,93 | 0,93 | 0,93 |
|  | IOP | 0,93 | 0,93 | 0,93 | 0,93 | 0,93 | 0,93 | 0,93 | 0,93 |  | 3,1 | 0,93 | 0,93 | 0,93 | 0,93 | 0,93 |
|  | MET | 0,93 | 3,1 | 3,1 | 0,93 | 0,93 | 0,93 | 0,93 | 0,93 | 0,93 |  | 0,93 | 1,51 | 0,93 | 0,93 | 0,93 |
|  | MEP | 0,93 | 3,1 | 3,1 | 1,51 | 0,93 | 0,93 | 0,93 | 0,93 | 0,93 | 3,1 |  | 1,51 | 0,93 | 0,93 | 0,93 |
|  | OXA | 0,93 | 1,51 | 0,93 | 0,93 | 0,93 | 0,93 | 0,93 | 0,93 | 0,93 | 0,93 | 0,93 |  | 0,93 | 0,93 | 0,93 |
|  | PHE | 0,93 | 1,51 | 0,93 | 0,93 | 0,93 | 0,93 | 1,51 | 0,93 | 0,93 | 1,51 | 3,1 | 1,51 |  | 0,93 | 0,93 |
|  | SUL | 0,93 | 0,93 | 0,93 | 0,93 | 0,93 | 0,93 | 0,93 | 0,93 | 0,93 | 3,1 | 0,93 | 0,93 | 0,93 |  | 0,93 |
|  | VAL | 3,1 | 0,93 | 0,93 | 1,51 | 5 | 3,1 | 0,93 | 1,51 | 0,93 | 0,93 | 1,51 | 0,93 | 3,1 | 0,93 |  |
| Pairwise interaction magnitude was attributed according to average internal exposure determined by Roden et al. (2015) and USFDA (2012)interaction ranges (*M_none_* = 0.93, *M_minor_* = 1.51, *M_moderate_* = 3.1, *M_major_* = 5). Pairwise symmetrical effects (2-way interactions) were not identified. AMA = amantadine. CBZ = carbamazepine. CIP = ciprofloxacin. CYC = cyclophosphamide. DCF = diclofenac. DOX = doxycycline. EE2 = 17α-ethinylestradiol. ERY = erythromycin. IOP = iopamidol. MEP = metoprolol. MET = metformin. OXA = oxazepam. PHE = phenazone. SUL = sulfamethazine. VAL = valsartan. | | | | | | | | | | | | | | | | |

# **TABLE S13.** Binary weight-of-evidence factor (*B*) of drug interactions between pairs of active pharmaceutical ingredient (API).

|  |  | Receptor | | | | | | | | | | | | | | |
| --- | --- | --- | --- | --- | --- | --- | --- | --- | --- | --- | --- | --- | --- | --- | --- | --- |
| Actor | API | EE2 | AMA | CBZ | CIP | CYC | DCF | DOX | ERY | IOP | MET | MEP | OXA | PHE | SUL | VAL |
|  | EE2 |  | 0 | 0 | 0 | 0 | -1 | 0 | 0 | 0 | -1 | 0 | 0 | 1 | 0 | 0 |
|  | AMA | 0 |  | 1 | 1 | 0 | 0 | 0 | 1 | 0 | 0 | 0 | 0 | 0 | 0 | 0 |
|  | CBZ | -1 | 0 |  | 0 | 0 | -1 | 0 | 0 | 0 | 0 | 0 | 1 | 1 | 0 | 0 |
|  | CIP | 1 | 0 | 1 |  | 1 | 1 | 0 | 1 | 0 | 1 | 0 | 1 | 1 | 0 | 0 |
|  | CYC | 1 | 0 | -1 | 0 |  | 1 | 0 | 0 | 0 | 0 | 0 | 0 | 1 | 0 | 0 |
|  | DCF | 0 | 1 | 0 | 0 | 0 |  | 1 | 1 | 0 | 1 | -1 | 1 | 1 | 0 | 0 |
|  | DOX | -1 | 1 | 1 | 1 | 1 | 0 |  | 0 | 0 | 1 | 1 | 1 | 0 | 0 | 0 |
|  | ERY | 1 | 0 | 1 | 0 | 1 | 0 | 0 |  | 0 | 0 | 0 | 0 | 0 | 0 | 0 |
|  | IOP | 0 | 0 | 0 | 0 | 0 | 0 | 0 | 0 |  | 1 | 0 | 0 | 0 | 0 | 0 |
|  | MET | 0 | 1 | 1 | 0 | 0 | 0 | 0 | 0 | 0 |  | 0 | 1 | 0 | 0 | 0 |
|  | MEP | 0 | 1 | 1 | 1 | 0 | 0 | 0 | 0 | 0 | 1 |  | 1 | 0 | 0 | 0 |
|  | OXA | 0 | 1 | 0 | 0 | 0 | 0 | 0 | 0 | 0 | 0 | 0 |  | 0 | 0 | 0 |
|  | PHE | 0 | 1 | 0 | 0 | 0 | 0 | 1 | 0 | 0 | 1 | -1 | 1 |  | 0 | 0 |
|  | SUL | 0 | 0 | 0 | 0 | 0 | 0 | 0 | 0 | 0 | 1 | 0 | 0 | 0 |  | 0 |
|  | VAL | 1 | 0 | 0 | 1 | 1 | 1 | 0 | 1 | 0 | 0 | 1 | 0 | 1 | 0 |  |
| Interaction effect information obtained from Drugbank Interaction Checker^©^ (Wishart et al. 2018) was assumed to be primarily derived from human data and the effects to be more than or less than additive. Pairwise symmetrical effects (2-way interactions) were not identified. AMA = amantadine; CBZ = carbamazepine. CIP = ciprofloxacin. CYC = cyclophosphamide. DCF = diclofenac. DOX = doxycycline. EE2 = 17α-ethinylestradiol. ERY = erythromycin. IOP = iopamidol. MEP = metoprolol. MET = metformin. OXA = oxazepam. PHE = phenazone. SUL = sulfamethazine. VAL = valsartan. | | | | | | | | | | | | | | | | |

# **TABLE S14.** Categorisation of drug interaction effects for all 210 pairs of active pharmaceutical ingredients in the present study.

| Interaction effect | Less than additive | Additive | Greater than additive |
| --- | --- | --- | --- |
| None | - | 147 (70%) | - |
| Minor | 1 (0.5%) | - | 21 (10%) |
| Moderate | 5 (2.4%) | - | 22 (10.5%) |
| Major | 2 (1%) | - | 12 (5.7%) |

**REFERENCES**

Baba H, Ueno Y, Hashida M, Yamashita F. 2017. Quantitative prediction of ionization effect on human skin permeability. International Journal of Pharmaceutics. 522(1):222-233.

Brown MB, Lau C-H, Lim ST, Sun Y, Davey N, Moss GP, Yoo S-H, De Muynck C. 2012. An evaluation of the potential of linear and nonlinear skin permeation models for the prediction of experimentally measured percutaneous drug absorption. Journal of Pharmacy and Pharmacology. 64(4):566-577.

Bull S, Green O, Carter J. 2014. Objective 6: Final report. Didcot, UK: Ricardo-AEA. No. Ricardo-AEA/R/ED59005.

Cal/EPA. 1992. Expedited cancer potency values and proposed regulatory levels for certain proposition 65 carcinogens. California Environmental Agency.

CBS. 2019. Lengte, onder- en overgewicht vanaf 1981. 2019 ed. CBS Open data StatLine.

Chen L-j, Lian G-p, Han L-j. 2007. Prediction of human skin permeability using artificial neural network (ANN) modeling. Acta Pharmacologica Sinica. 28(4):591-600.

Cheng F, Li W, Zhou Y, Shen J, Wu Z, Liu G, Lee PW, Tang Y. 2012. admetSAR: A Comprehensive Source and Free Tool for Assessment of Chemical ADMET Properties. Journal of Chemical Information and Modeling. 52(11):3099-3105.

Cunningham VL, Perino C, D'Aco VJ, Hartmann A, Bechter R. 2010. Human health risk assessment of carbamazepine in surface waters of North America and Europe. Regulatory toxicology and pharmacology : RTP. 56(3):343-351.

Daina A, Michielin O, Zoete V. 2017. SwissADME: a free web tool to evaluate pharmacokinetics, drug-likeness and medicinal chemistry friendliness of small molecules. Scientific Reports. 7(1):42717.

Fourie L, Breytenbach JC, Du Plessis J, Goosen C, Swart H, Hadgraft J. 2004. Percutaneous delivery of carbamazepine and selected N-alkyl and N-hydroxyalkyl analogues. International Journal of Pharmaceutics. 279(1):59-66.

Fredriks AM, van Buuren S, Burgmeijer RJF, Meulmeester JF, Beuker RJ, Brugman E, Roede MJ, Verloove-Vanhorick SP, Wit J-M. 2000. Continuing Positive Secular Growth Change in the Netherlands 1955–1997. Pediatric Research. 47(3):316-323.

Hou T, Wang J, Li Y. 2007. ADME Evaluation in Drug Discovery. 8. The Prediction of Human Intestinal Absorption by a Support Vector Machine. Journal of Chemical Information and Modeling. 47(6):2408-2415.

Kumar A, Chang B, Xagoraraki I. 2010. Human health risk assessment of pharmaceuticals in water: issues and challenges ahead. Int J Environ Res Public Health. 7(11):3929-3953.

Kumar A, Xagoraraki I. 2010. Human health risk assessment of pharmaceuticals in water: An uncertainty analysis for meprobamate, carbamazepine, and phenytoin. Regulatory Toxicology and Pharmacology. 57(2):146-156.

Lee J-Y, Choi J-W, Kim H. 2008. Determination of Body Surface Area and Formulas to Estimate Body Surface Area Using the Alginate Method. Journal of PHYSIOLOGICAL ANTHROPOLOGY. 27(2):71-82.

Lee PH, Conradi R, Shanmugasundaram V. 2010. Development of an in silico model for human skin permeation based on a Franz cell skin permeability assay. Bioorganic & Medicinal Chemistry Letters. 20(1):69-73.

Lian G, Chen L, Han L. 2008. An evaluation of mathematical models for predicting skin permeability. Journal of Pharmaceutical Sciences. 97(1):584-598.

Livingston EH, Lee S. 2000. Percentage of Burned Body Surface Area Determination in Obese and Nonobese Patients. Journal of Surgical Research. 91(2):106-110.

Manallack DT. 2007. The pK(a) Distribution of Drugs: Application to Drug Discovery. Perspectives in medicinal chemistry. 1:25-38.

Modamio P, Lastra CF, Mariño EL. 2000. A comparative in vitro study of percutaneous penetration of β-blockers in human skin. International Journal of Pharmaceutics. 194(2):249-259.

Palm K, Stenberg P, Luthman K, Artursson P. 1997. Polar Molecular Surface Properties Predict the Intestinal Absorption of Drugs in Humans. Pharmaceutical Research. 14(5):568-571.

Potts RO, Guy RH. 1992. Predicting Skin Permeability. Pharmaceutical Research. 9(5):663-669.

RIVM. 2018. Dutch National Consumption Survey 2012-2016.

Roden NM, Sargent EV, DiFerdinando GT, Hong J-Y, Robson MG. 2015. The Cumulative Risk to Human Health of Pharmaceuticals in New Jersey Surface Water. Human and Ecological Risk Assessment: An International Journal. 21(1):280-295.

Schets FM, Schijven JF, de Roda Husman AM. 2011. Exposure assessment for swimmers in bathing waters and swimming pools. Water Research. 45(7):2392-2400.

Schriks M, Heringa MB, van der Kooi MME, de Voogt P, van Wezel AP. 2010. Toxicological relevance of emerging contaminants for drinking water quality. Water research. 44(2):461-476.

Schwab BW, Hayes EP, Fiori JM, Mastrocco FJ, Roden NM, Cragin D, Meyerhoff RD, D’Aco VJ, Anderson PD. 2005. Human pharmaceuticals in US surface waters: A human health risk assessment. Regulatory Toxicology and Pharmacology. 42(3):296-312.

Shen J, Cheng F, Xu Y, Li W, Tang Y. 2010. Estimation of ADME Properties with Substructure Pattern Recognition. Journal of Chemical Information and Modeling. 50(6):1034-1041.

Suchomel A, Goeden H, Dady J, Shubat P. 2015. Pharmaceutical Water Screening Values Report. St Paul, Minnesota: Minnesota Department of Health.

USFDA. 2012. Draft: Guidance for Industry: Drug Interaction Studies—Study Design, Data Analysis, Implications for Dosing, and Labeling Recommendations. Center for Drug Evaluation and Research, Washington, DC, USA.

Williams ES, Brooks BW. 2012. Human Health Risk Assessment for Pharmaceuticals in the Environment: Existing Practice, Uncertainty, and Future Directions. In: Brooks BW, Huggett DB, editors. Human Pharmaceuticals in the Environment. 1 ed. New York: Springer-Verlag. p. 304.

Wishart DS, Feunang YD, Guo AC, Lo EJ, Marcu A, Grant JR, Sajed T, Johnson D, Li C, Sayeeda Z et al. 2018. DrugBank 5.0: a major update to the DrugBank database for 2018. Nucleic Acids Res. 46(D1):D1074-d1082.
