# Supplementary material for: Human health risk assessment of pharmaceuticals in the European Vecht River
Source: Integr Environ Assess Manag. 2022 Feb 28;18(6):1639–54. doi: 10.1002/ieam.4588 (PMC9790459; doi:10.1002/ieam.4588)
Supplement: Supplementary file 3 [file IEAM-18-1639-s003.docx]

**SUPPORTING INFORMATION**

**TITLE**

Human Health Risk Assessment of Pharmaceuticals in the European Vecht River

**AUTHORS**

Daniel J. Duarte ^a^, Rik Oldenkamp ^b^, Ad M. J. Ragas ^a, c^

*^a^ Radboud University Nijmegen, Institute for Water & Wetland Research, Department of Environmental Science, PO Box 9010, NL-6500 GL, Nijmegen, The Netherlands*

*^b^ Department of Global Health-Amsterdam Institute for Global Health and Development, Amsterdam UMC, University of Amsterdam, Paasheuvelweg 25, NL-1105 BP, Amsterdam, The Netherlands*

*^c^ Open University, Faculty of Science, Department of Environmental Sciences,* *Valkenburgerweg 177, 6419 AT, Heerlen, The Netherlands*

Hazard Quotient ($HQ$) can be renamed as Hazard Quotient target (${HQ}_{t}$):

$${HQ}_{t}=\frac{U_{t}}{ISD}\Leftrightarrow$$

$${HQ}_{t}\cdot ISD=U_{t}\Leftrightarrow$$

The expression can be rewritten in its expanded form according to Table 2 as:

$${HQ}_{t}\cdot ISD=\sum_{i=1}^{n} \left( \frac{y_{i}}{y_{lt}}U_{i} \right)\Leftrightarrow$$

$${HQ}_{t}\cdot ISD=\sum_{i=1}^{n} \left( \frac{y_{i}}{y_{lt}}{(U}_{oral,i}+U_{dermal,i}) \right)\Leftrightarrow$$

$${HQ}_{t}\cdot ISD=\left( \frac{1}{80}{(U}_{oral, 0-1}+U_{dermal,0-1}) \right)+\left( \frac{4}{80}{(U}_{oral,1-5}+U_{dermal,1-5}) \right)+\left( \frac{5}{80}(U_{oral,5-10}+U_{dermal,5-10}) \right)+\left( \frac{8}{80}{(U}_{oral,10-18}+U_{dermal,10-18}) \right)+\left( \frac{62}{80}(U_{oral,18-80}+U_{dermal,18-80}) \right)\Leftrightarrow$$

$${HQ}_{t}\cdot ISD=\left( \frac{1}{80}\left( U_{os}+U_{dw}+U_{f}+U_{dermal} \right) \right)+\left( \frac{4}{80}{(U}_{os}+U_{dw}+U_{f}+U_{dermal}) \right)+\left( \frac{5}{80}(U_{os}+U_{dw}+U_{f}+U_{dermal}) \right)+\left( \frac{8}{80}{(U}_{os}+U_{dw}+U_{f}+U_{dermal}) \right)+\left( \frac{62}{80}{(U}_{os}+U_{dw}+U_{f}+U_{dermal}) \right)\Leftrightarrow$$

$${HQ}_{t}\cdot ISD=\left( \frac{1}{80}\left( \frac{q_{s}\cdot t_{e}\cdot s_{e}\cdot f_{GI}\cdot C_{w}}{d\cdot m}+\frac{q_{w}\cdot f_{GI}\cdot C_{dw}}{m}+\frac{q_{f}\cdot f_{GI}\cdot C_{f}}{m}+\frac{A_{s}\cdot f_{s}\cdot k_{p}\cdot t_{e}\cdot s_{e}\cdot C_{w}}{d\cdot m} \right) \right)+\left( \frac{4}{80}\left( \frac{q_{s}\cdot t_{e}\cdot s_{e}\cdot f_{GI}\cdot C_{w}}{d\cdot m}+\frac{q_{w}\cdot f_{GI}\cdot C_{dw}}{m}+\frac{q_{f}\cdot f_{GI}\cdot C_{f}}{m}+\frac{A_{s}\cdot f_{s}\cdot k_{p}\cdot t_{e}\cdot s_{e}\cdot C_{w}}{d\cdot m} \right) \right)+\left( \frac{5}{80}\left( \frac{q_{s}\cdot t_{e}\cdot s_{e}\cdot f_{GI}\cdot C_{w}}{d\cdot m}+\frac{q_{w}\cdot f_{GI}\cdot C_{dw}}{m}+\frac{q_{f}\cdot f_{GI}\cdot C_{f}}{m}+\frac{A_{s}\cdot f_{s}\cdot k_{p}\cdot t_{e}\cdot s_{e}\cdot C_{w}}{d\cdot m} \right) \right)+\left( \frac{8}{80}\left( \frac{q_{s}\cdot t_{e}\cdot s_{e}\cdot f_{GI}\cdot C_{w}}{d\cdot m}+\frac{q_{w}\cdot f_{GI}\cdot C_{dw}}{m}+\frac{q_{f}\cdot f_{GI}\cdot C_{f}}{m}+\frac{A_{s}\cdot f_{s}\cdot k_{p}\cdot t_{e}\cdot s_{e}\cdot C_{w}}{d\cdot m} \right) \right)+\left( \frac{62}{80}\left( \frac{q_{s}\cdot t_{e}\cdot s_{e}\cdot f_{GI}\cdot C_{w}}{d\cdot m}+\frac{q_{w}\cdot f_{GI}\cdot C_{dw}}{m}+\frac{q_{f}\cdot f_{GI}\cdot C_{f}}{m}+\frac{A_{s}\cdot f_{s}\cdot k_{p}\cdot t_{e}\cdot s_{e}\cdot C_{w}}{d\cdot m} \right) \right)\Leftrightarrow$$

$${HQ}_{t}\cdot ISD=\left( 0.0125\left( \frac{q_{s}\cdot t_{e}\cdot s_{e}\cdot f_{GI}\cdot C_{w}}{d\cdot m}+\frac{q_{w}\cdot f_{GI}\cdot C_{dw}}{m}+\frac{q_{f}\cdot f_{GI}\cdot\left( C_{w}\cdot BCF \right)}{m}+\frac{A_{s}\cdot f_{s}\cdot k_{p}\cdot t_{e}\cdot s_{e}\cdot C_{w}}{d\cdot m} \right) \right)+\left( 0.05\left( \frac{q_{s}\cdot t_{e}\cdot s_{e}\cdot f_{GI}\cdot C_{w}}{d\cdot m}+\frac{q_{w}\cdot f_{GI}\cdot C_{dw}}{m}+\frac{q_{f}\cdot f_{GI}\cdot\left( C_{w}\cdot BCF \right)}{m}+\frac{A_{s}\cdot f_{s}\cdot k_{p}\cdot t_{e}\cdot s_{e}\cdot C_{w}}{d\cdot m} \right) \right)+\left( 0.0625\left( \frac{q_{s}\cdot t_{e}\cdot s_{e}\cdot f_{GI}\cdot C_{w}}{d\cdot m}+\frac{q_{w}\cdot f_{GI}\cdot C_{dw}}{m}+\frac{q_{f}\cdot f_{GI}\cdot\left( C_{w}\cdot BCF \right)}{m}+\frac{A_{s}\cdot f_{s}\cdot k_{p}\cdot t_{e}\cdot s_{e}\cdot C_{w}}{d\cdot m} \right) \right)+\left( 0.1\left( \frac{q_{s}\cdot t_{e}\cdot s_{e}\cdot f_{GI}\cdot C_{w}}{d\cdot m}+\frac{q_{w}\cdot f_{GI}\cdot C_{dw}}{m}+\frac{q_{f}\cdot f_{GI}\cdot\left( C_{w}\cdot BCF \right)}{m}+\frac{A_{s}\cdot f_{s}\cdot k_{p}\cdot t_{e}\cdot s_{e}\cdot C_{w}}{d\cdot m} \right) \right)+\left( 0.775\left( \frac{q_{s}\cdot t_{e}\cdot s_{e}\cdot f_{GI}\cdot C_{w}}{d\cdot m}+\frac{q_{w}\cdot f_{GI}\cdot C_{dw}}{m}+\frac{q_{f}\cdot f_{GI}\cdot\left( C_{w}\cdot BCF \right)}{m}+\frac{A_{s}\cdot f_{s}\cdot k_{p}\cdot t_{e}\cdot s_{e}\cdot C_{w}}{d\cdot m} \right) \right)\Leftrightarrow$$

$${HQ}_{t}\cdot ISD=\left( 0.0125\left( \frac{q_{s}\cdot t_{e}\cdot s_{e}\cdot f_{GI}\cdot C_{w}+{d\cdot q}_{w}\cdot f_{GI}\cdot C_{dw}+d\cdot q_{f}\cdot f_{GI}\cdot C_{w}\cdot BCF+A_{s}\cdot f_{s}\cdot k_{p}\cdot t_{e}\cdot s_{e}\cdot C_{w}}{d\cdot m_{1}} \right) \right)+\left( 0.05\left( \frac{q_{s}\cdot t_{e}\cdot s_{e}\cdot f_{GI}\cdot C_{w}+{d\cdot q}_{w}\cdot f_{GI}\cdot C_{dw}+d\cdot q_{f}\cdot f_{GI}\cdot C_{w}\cdot BCF+A_{s}\cdot f_{s}\cdot k_{p}\cdot t_{e}\cdot s_{e}\cdot C_{w}}{d\cdot m_{2}} \right) \right)+\left( 0.0625\left( \frac{q_{s}\cdot t_{e}\cdot s_{e}\cdot f_{GI}\cdot C_{w}+{d\cdot q}_{w}\cdot f_{GI}\cdot C_{dw}+d\cdot q_{f}\cdot f_{GI}\cdot C_{w}\cdot BCF+A_{s}\cdot f_{s}\cdot k_{p}\cdot t_{e}\cdot s_{e}\cdot C_{w}}{d\cdot m_{3}} \right) \right)+\left( 0.1\left( \frac{q_{s}\cdot t_{e}\cdot s_{e}\cdot f_{GI}\cdot C_{w}+{d\cdot q}_{w}\cdot f_{GI}\cdot C_{dw}+d\cdot q_{f}\cdot f_{GI}\cdot C_{w}\cdot BCF+A_{s}\cdot f_{s}\cdot k_{p}\cdot t_{e}\cdot s_{e}\cdot C_{w}}{d\cdot m_{4}} \right) \right)+\left( 0.775\left( \frac{q_{s}\cdot t_{e}\cdot s_{e}\cdot f_{GI}\cdot C_{w}+{d\cdot q}_{w}\cdot f_{GI}\cdot C_{dw}+d\cdot q_{f}\cdot f_{GI}\cdot C_{w}\cdot BCF+A_{s}\cdot f_{s}\cdot k_{p}\cdot t_{e}\cdot s_{e}\cdot C_{w}}{d\cdot m_{5}} \right) \right)$$

Let:

$$Z=q_{s}\cdot t_{e}\cdot s_{e}\cdot f_{GI}$$

$$W={d\cdot q}_{w}\cdot f_{GI}\cdot C_{dw}$$

$$Y=d\cdot q_{f}\cdot f_{GI}\cdot BCF$$

$$X=A_{s}\cdot f_{s}\cdot k_{p}\cdot t_{e}\cdot s_{e}$$

$$k_{1}=0.0125$$

$$k_{2}=0.05$$

$$k_{3}=0.0625$$

$$k_{4}=0.1$$

$$k_{5}=0.775$$

Substituting:

$${HQ}_{t}\cdot ISD=k_{1}\left( \frac{Z_{1}\cdot C_{w}+W_{1}+Y_{1}\cdot C_{w}+X_{1}\cdot C_{w}}{d\cdot m_{1}} \right)+k_{2}\left( \frac{Z_{2}\cdot C_{w}+W_{2}+Y_{2}\cdot C_{w}+X_{2}\cdot C_{w}}{d\cdot m_{2}} \right)+k_{3}\left( \frac{Z_{3}\cdot C_{w}+W_{3}+Y_{3}\cdot C_{w}+X_{3}\cdot C_{w}}{d\cdot m_{3}} \right)+k_{4}\left( \frac{Z_{4}\cdot C_{w}+W_{4}+Y_{4}\cdot C_{w}+X_{4}\cdot C_{w}}{d\cdot m_{4}} \right)+k_{5}\left( \frac{Z_{5}\cdot C_{w}+W_{5}+Y_{5}\cdot C_{w}+X_{5}\cdot C_{w}}{d\cdot m_{5}} \right)\Leftrightarrow$$

$${HQ}_{t}\cdot ISD=k_{1}\left( \frac{Z_{1}\cdot C_{w}+W_{1}+Y_{1}\cdot C_{w}+X_{1}\cdot C_{w}}{d\cdot m_{1}} \right)+k_{2}\left( \frac{Z_{2}\cdot C_{w}+W_{2}+Y_{2}\cdot C_{w}+X_{2}\cdot C_{w}}{d\cdot m_{2}} \right)+k_{3}\left( \frac{Z_{3}\cdot C_{w}+W_{3}+Y_{3}\cdot C_{w}+X_{3}\cdot C_{w}}{d\cdot m_{3}} \right)+k_{4}\left( \frac{Z_{4}\cdot C_{w}+W_{4}+Y_{4}\cdot C_{w}+X_{4}\cdot C_{w}}{d\cdot m_{4}} \right)+k_{5}\left( \frac{Z_{5}\cdot C_{w}+W_{5}+Y_{5}\cdot C_{w}+X_{5}\cdot C_{w}}{d\cdot m_{5}} \right)\Leftrightarrow$$

$${HQ}_{t}\cdot ISD\cdot d=k_{1}\left( \frac{Z_{1}\cdot C_{w}+W_{1}+Y_{1}\cdot C_{w}+X_{1}\cdot C_{w}}{m_{1}} \right)+k_{2}\left( \frac{Z_{2}\cdot C_{w}+W_{2}+Y_{2}\cdot C_{w}+X_{2}\cdot C_{w}}{m_{2}} \right)+k_{3}\left( \frac{Z_{3}\cdot C_{w}+W_{3}+Y_{3}\cdot C_{w}+X_{3}\cdot C_{w}}{m_{3}} \right)+k_{4}\left( \frac{Z_{4}\cdot C_{w}+W_{4}+Y_{4}\cdot C_{w}+X_{4}\cdot C_{w}}{m_{4}} \right)+k_{5}\left( \frac{Z_{5}\cdot C_{w}+W_{5}+Y_{5}\cdot C_{w}+X_{5}\cdot C_{w}}{m_{5}} \right)\Leftrightarrow$$

$${HQ}_{t}\cdot ISD\cdot d=\frac{{k_{1}Z}_{1}\cdot C_{w}+k_{1}W_{1}+{k_{1}Y}_{1}\cdot C_{w}+{k_{1}X}_{1}\cdot C_{w}}{m_{1}}+\frac{{k_{2}Z}_{2}\cdot C_{w}+{k_{2}W}_{2}+{k_{2}Y}_{2}\cdot C_{w}+{k_{2}X}_{2}\cdot C_{w}}{m_{2}}+\frac{{k_{3}Z}_{3}\cdot C_{w}+{k_{3}W}_{3}+{k_{3}Y}_{3}\cdot C_{w}+{k_{3}X}_{3}\cdot C_{w}}{m_{3}}+\frac{{k_{4}Z}_{4}\cdot C_{w}+{k_{4}W}_{4}+{k_{4}Y}_{4}\cdot C_{w}+{k_{4}X}_{4}\cdot C_{w}}{m_{4}}+\frac{{k_{5}Z}_{5}\cdot C_{w}+k_{5}W_{5}+{k_{5}Y}_{5}\cdot C_{w}+{k_{5}X}_{5}\cdot C_{w}}{m_{5}}\Leftrightarrow$$

$${HQ}_{t}\cdot ISD\cdot d=\frac{{{m_{2}m_{3}m_{4}m_{5}(k}_{1}Z}_{1}\cdot C_{w}+k_{1}W_{1}+{k_{1}Y}_{1}\cdot C_{w}+{k_{1}X}_{1}\cdot C_{w})}{m_{1}m_{2}m_{3}m_{4}m_{5}}+\frac{m_{1}m_{3}m_{4}m_{5}({k_{2}Z}_{2}\cdot C_{w}+{k_{2}W}_{2}+{k_{2}Y}_{2}\cdot C_{w}+{k_{2}X}_{2}\cdot C_{w})}{m_{1}m_{2}m_{3}m_{4}m_{5}}+\frac{m_{1}m_{2}m_{4}m_{5}({k_{3}Z}_{3}\cdot C_{w}+{k_{3}W}_{3}+{k_{3}Y}_{3}\cdot C_{w}+{k_{3}X}_{3}\cdot C_{w})}{m_{1}m_{2}m_{3}m_{4}m_{5}}+\frac{{{m_{1}m_{2}m_{3}m_{5}(k}_{4}Z}_{4}\cdot C_{w}+{k_{4}W}_{4}+{k_{4}Y}_{4}\cdot C_{w}+{k_{4}X}_{4}\cdot C_{w})}{m_{1}m_{2}m_{3}m_{4}m_{5}}+\frac{m_{1}m_{2}m_{3}m_{4}({k_{5}Z}_{5}\cdot C_{w}+k_{5}W_{5}+{k_{5}Y}_{5}\cdot C_{w}+{k_{5}X}_{5}\cdot C_{w})}{m_{1}m_{2}m_{3}m_{4}m_{5}}\Leftrightarrow$$

$${HQ}_{t}\cdot ISD\cdot d=\frac{{{m_{2}m_{3}m_{4}m_{5}(k}_{1}Z}_{1}\cdot C_{w}+k_{1}W_{1}+{k_{1}Y}_{1}\cdot C_{w}+{k_{1}X}_{1}\cdot C_{w})+m_{1}m_{3}m_{4}m_{5}({k_{2}Z}_{2}\cdot C_{w}+{k_{2}W}_{2}+{k_{2}Y}_{2}\cdot C_{w}+{k_{2}X}_{2}\cdot C_{w})+m_{1}m_{2}m_{4}m_{5}({k_{3}Z}_{3}\cdot C_{w}+{k_{3}W}_{3}+{k_{3}Y}_{3}\cdot C_{w}+{k_{3}X}_{3}\cdot C_{w})+{{m_{1}m_{2}m_{3}m_{5}(k}_{4}Z}_{4}\cdot C_{w}+{k_{4}W}_{4}+{k_{4}Y}_{4}\cdot C_{w}+{k_{4}X}_{4}\cdot C_{w})+m_{1}m_{2}m_{3}m_{4}({k_{5}Z}_{5}\cdot C_{w}+k_{5}W_{5}+{k_{5}Y}_{5}\cdot C_{w}+{k_{5}X}_{5}\cdot C_{w})}{m_{1}m_{2}m_{3}m_{4}m_{5}}$$

Let:

$$M_{-1}=\sum_{i=2}^{5} m_{i}$$

$$M_{-2}=\sum_{i\in S} m_{i}, for S=\left\{ 1, 3, 4, 5 \right\}$$

$$M_{-3}=\sum_{i\in S} m_{i}, for S=\left\{ 1, 2, 4, 5 \right\}$$

$$M_{-4}=\sum_{i\in S} m_{i}, for S=\left\{ 1, 2, 3, 5 \right\}$$

$$M_{-5}=\sum_{i\in S} m_{i}, for S=\left\{ 1, 2, 3, 4 \right\}$$

$$M_{p}=\sum_{i=1}^{5} m_{i}$$

Substituting:

$${HQ}_{t}\cdot ISD\cdot d=\frac{{{M_{-1}(k}_{1}Z}_{1}\cdot C_{w}+k_{1}W_{1}+{k_{1}Y}_{1}\cdot C_{w}+{k_{1}X}_{1}\cdot C_{w})+M_{-2}({k_{2}Z}_{2}\cdot C_{w}+{k_{2}W}_{2}+{k_{2}Y}_{2}\cdot C_{w}+{k_{2}X}_{2}\cdot C_{w})+M_{-3}({k_{3}Z}_{3}\cdot C_{w}+{k_{3}W}_{3}+{k_{3}Y}_{3}\cdot C_{w}+{k_{3}X}_{3}\cdot C_{w})+{{M_{-4}(k}_{4}Z}_{4}\cdot C_{w}+{k_{4}W}_{4}+{k_{4}Y}_{4}\cdot C_{w}+{k_{4}X}_{4}\cdot C_{w})+M_{-5}({k_{5}Z}_{5}\cdot C_{w}+k_{5}W_{5}+{k_{5}Y}_{5}\cdot C_{w}+{k_{5}X}_{5}\cdot C_{w})}{M_{p}}\Leftrightarrow$$

$${HQ}_{t}\cdot ISD\cdot d\cdot M_{p}={{M_{-1}k}_{1}Z}_{1}\cdot C_{w}+{M_{-1}k}_{1}W_{1}+{M_{-1}k_{1}Y}_{1}\cdot C_{w}+{M_{-1}k_{1}X}_{1}\cdot C_{w}+M_{-2}{k_{2}Z}_{2}\cdot C_{w}+{M_{-2}k_{2}W}_{2}+{M_{-2}k_{2}Y}_{2}\cdot C_{w}+{{M_{-2}k}_{2}X}_{2}\cdot C_{w}+M_{-3}{k_{3}Z}_{3}\cdot C_{w}+{{M_{-3}k}_{3}W}_{3}+M_{-3}{k_{3}Y}_{3}\cdot C_{w}+{{M_{-3}k}_{3}X}_{3}\cdot C_{w}+{{M_{-4}k}_{4}Z}_{4}\cdot C_{w}+{{M_{-4}k}_{4}W}_{4}+{{M_{-4}k}_{4}Y}_{4}\cdot C_{w}+{M_{-4}k_{4}X}_{4}\cdot C_{w}+M_{-5}{k_{5}Z}_{5}\cdot C_{w}+{M_{-5}k}_{5}W_{5}+{{M_{-5}k}_{5}Y}_{5}\cdot C_{w}+{M_{-5}k_{5}X}_{5}\cdot C_{w}\Leftrightarrow$$

$${HQ}_{t}\cdot ISD\cdot d\cdot M_{p}-{M_{-1}k}_{1}W_{1}-{M_{-2}k_{2}W}_{2}-{{M_{-3}k}_{3}W}_{3}-{{M_{-4}k}_{4}W}_{4}-{M_{-5}k}_{5}W_{5}={{M_{-1}k}_{1}Z}_{1}\cdot C_{w}+{M_{-1}k_{1}Y}_{1}\cdot C_{w}+{M_{-1}k_{1}X}_{1}\cdot C_{w}+M_{-2}{k_{2}Z}_{2}\cdot C_{w}+{M_{-2}k_{2}Y}_{2}\cdot C_{w}+{{M_{-2}k}_{2}X}_{2}\cdot C_{w}+M_{-3}{k_{3}Z}_{3}\cdot C_{w}+M_{-3}{k_{3}Y}_{3}\cdot C_{w}+{{M_{-3}k}_{3}X}_{3}\cdot C_{w}+{{M_{-4}k}_{4}Z}_{4}\cdot C_{w}+{{M_{-4}k}_{4}Y}_{4}\cdot C_{w}+{M_{-4}k_{4}X}_{4}\cdot C_{w}+M_{-5}{k_{5}Z}_{5}\cdot C_{w}+{{M_{-5}k}_{5}Y}_{5}\cdot C_{w}+{M_{-5}k_{5}X}_{5}\cdot C_{w}\Leftrightarrow$$

$${HQ}_{t}\cdot ISD\cdot d\cdot M_{p}-{M_{-1}k}_{1}W_{1}-{M_{-2}k_{2}W}_{2}-{{M_{-3}k}_{3}W}_{3}-{{M_{-4}k}_{4}W}_{4}-{M_{-5}k}_{5}W_{5}={C_{w}({M_{-1}k}_{1}Z}_{1}+{M_{-1}k_{1}Y}_{1}+{M_{-1}k_{1}X}_{1}+M_{-2}{k_{2}Z}_{2}+{M_{-2}k_{2}Y}_{2}+{{M_{-2}k}_{2}X}_{2}+M_{-3}{k_{3}Z}_{3}+M_{-3}{k_{3}Y}_{3}+{{M_{-3}k}_{3}X}_{3}+{{M_{-4}k}_{4}Z}_{4}+{{M_{-4}k}_{4}Y}_{4}+{M_{-4}k_{4}X}_{4}+M_{-5}{k_{5}Z}_{5}+{{M_{-5}k}_{5}Y}_{5}+{M_{-5}k_{5}X}_{5})\Leftrightarrow$$

$$C_{w}=\frac{{HQ}_{t}\cdot ISD\cdot d\cdot M_{p}-{M_{-1}k}_{1}W_{1}-{M_{-2}k_{2}W}_{2}-{{M_{-3}k}_{3}W}_{3}-{{M_{-4}k}_{4}W}_{4}-{M_{-5}k}_{5}W_{5}}{{M_{-1}k}_{1}Z_{1}+{M_{-1}k_{1}Y}_{1}+{M_{-1}k_{1}X}_{1}+M_{-2}{k_{2}Z}_{2}+{M_{-2}k_{2}Y}_{2}+{{M_{-2}k}_{2}X}_{2}+M_{-3}{k_{3}Z}_{3}+M_{-3}{k_{3}Y}_{3}+{{M_{-3}k}_{3}X}_{3}+{{M_{-4}k}_{4}Z}_{4}+{{M_{-4}k}_{4}Y}_{4}+{M_{-4}k_{4}X}_{4}+M_{-5}{k_{5}Z}_{5}+{{M_{-5}k}_{5}Y}_{5}+{M_{-5}k_{5}X}_{5}}\Leftrightarrow$$

$$C_{w}=\frac{{HQ}_{t}\cdot ISD\cdot d\cdot M_{p}-{M_{-1}k}_{1}(W_{1})-{M_{-2}k_{2}(W}_{2})-{{M_{-3}k}_{3}(W}_{3})-{{M_{-4}k}_{4}(W}_{4})-{M_{-5}k}_{5}(W_{5})}{M_{-1}k_{1}(Z_{1}+Y_{1}+X_{1})+M_{-2}{k_{2}(Z}_{2}+Y_{2}+X_{2})+M_{-3}{k_{3}(Z}_{3}+Y_{3}+X_{3})+{{M_{-4}k}_{4}(Z}_{4}+Y_{4}+X_{4})+M_{-5}{k_{5}(Z}_{5}+Y_{5}+X_{5})}\Leftrightarrow$$

$$C_{w}=\frac{{HQ}_{t}\cdot ISD\cdot d\cdot M_{p}-{M_{-1}k}_{1}(W_{1})-{M_{-2}k_{2}(W}_{2})-{{M_{-3}k}_{3}(W}_{3})-{{M_{-4}k}_{4}(W}_{4})-{M_{-5}k}_{5}(W_{5})}{M_{-1}k_{1}(Z_{1}+d\cdot{q_{f}}_{1}\cdot f_{GI}\cdot BCF+X_{1})+M_{-2}{k_{2}(Z}_{2}+d\cdot{q_{f}}_{2}\cdot f_{GI}\cdot BCF+X_{2})+M_{-3}{k_{3}(Z}_{3}+d\cdot{q_{f}}_{3}\cdot f_{GI}\cdot BCF+X_{3})+{{M_{-4}k}_{4}(Z}_{4}+d\cdot{q_{f}}_{4}\cdot f_{GI}\cdot BCF+X_{4})+M_{-5}{k_{5}(Z}_{5}+d\cdot{q_{f}}_{5}\cdot f_{GI}\cdot BCF+X_{5})}$$

Let:

$$Y^{'}=d\cdot f_{GI}\cdot BCF$$

Substituting:

$$C_{w}=\frac{{HQ}_{t}\cdot ISD\cdot d\cdot M_{p}-{M_{-1}k}_{1}(W_{1})-{M_{-2}k_{2}(W}_{2})-{{M_{-3}k}_{3}(W}_{3})-{{M_{-4}k}_{4}(W}_{4})-{M_{-5}k}_{5}(W_{5})}{M_{-1}k_{1}(Z_{1}+Y^{'}\cdot{q_{f}}_{1}+X_{1})+M_{-2}{k_{2}(Z}_{2}+Y^{'}\cdot{q_{f}}_{2}+X_{2})+M_{-3}{k_{3}(Z}_{3}+Y^{'}\cdot{q_{f}}_{3}+X_{3})+{{M_{-4}k}_{4}(Z}_{4}+Y^{'}\cdot{q_{f}}_{4}+X_{4})+M_{-5}{k_{5}(Z}_{5}+Y^{'}\cdot{q_{f}}_{5}+X_{5})}\Leftrightarrow$$

$$C_{w}=\frac{{HQ}_{t}\cdot ISD\cdot d\cdot M_{p}-{M_{-1}k}_{1}(W_{1})-{M_{-2}k_{2}(W}_{2})-{{M_{-3}k}_{3}(W}_{3})-{{M_{-4}k}_{4}(W}_{4})-{M_{-5}k}_{5}(W_{5})}{M_{-1}k_{1}Z_{1}+M_{-1}k_{1}Y^{'}\cdot{q_{f}}_{1}+{M_{-1}k_{1}X}_{1}+M_{-2}k_{2}Z_{2}+M_{-2}k_{2}Y^{'}\cdot{q_{f}}_{2}+M_{-2}k_{2}X_{2}+M_{-3}k_{3}Z_{3}+M_{-3}k_{3}Y^{'}\cdot{q_{f}}_{3}+M_{-3}k_{3}X_{3}+{{M_{-4}k}_{4}Z}_{4}+{M_{-4}k}_{4}Y^{'}\cdot{q_{f}}_{4}+{M_{-4}k}_{4}X_{4}+M_{-5}k_{5}Z_{5}+M_{-5}k_{5}Y^{'}\cdot{q_{f}}_{5}+M_{-5}k_{5}X_{5}}$$

Let:

$$G_{i}=\sum_{i=1}^{5} \left( M_{-i}k_{i}\left( Z_{i}+X_{i} \right) \right)$$

$$F_{i}=M_{-i}k_{i}Y^{'}$$

$$A={HQ}_{t}\cdot ISD\cdot d\cdot M_{p}-{M_{-1}k}_{1}(W_{1})-{M_{-2}k_{2}(W}_{2})-{{M_{-3}k}_{3}(W}_{3})-{{M_{-4}k}_{4}(W}_{4})-{M_{-5}k}_{5}(W_{5})$$

Substituting:

$$C_{w}=\frac{A}{G_{1}+F_{1}\cdot{q_{f}}_{1}+G_{2}+F_{2}\cdot{q_{f}}_{2}+G_{3}+F_{3}\cdot{q_{f}}_{3}+G_{4}+F_{4}\cdot{q_{f}}_{4}+G_{5}+F_{5}\cdot{q_{f}}_{5}}$$

Let an arbitrary weighting factor $n$ be defined by $q_{r}=60 000 mg/day$. This is an operation which allows factorization later on; it has no impact on the final output, so $q_{r}$ could take any value. Thus, ${q_{f}}_{i}=n_{i}q_{r}$.

$$n_{1}=0$$

$$n_{2}=0.87667$$

$$n_{3}=1.166$$

$$n_{4}=1.12917$$

$$n_{5}=1.81615$$

Substituting:

$$C_{w}=\frac{A}{G_{1}+F_{1}\cdot n_{1}q_{r}+G_{2}+F_{2}\cdot n_{2}q_{r}+G_{3}+F_{3}\cdot n_{3}q_{r}+G_{4}+F_{4}\cdot n_{4}q_{r}+G_{5}+F_{5}\cdot n_{5}q_{r}}\Leftrightarrow$$

$$C_{w}=\frac{A}{\left( G_{1}+G_{2}+G_{3}+G_{4}+G_{5} \right)+(F_{1}n_{1}+F_{2}n_{2}+F_{3}n_{3}+F_{4}n_{4}+F_{5}n_{5})q_{r}}\Leftrightarrow$$

$$C_{w}=\frac{A}{\left( G_{1}+G_{2}+G_{3}+G_{4}+G_{5} \right)+(F_{1}n_{1}+F_{2}n_{2}+F_{3}n_{3}+F_{4}n_{4}+F_{5}n_{5})q_{r}}$$

Let:

$$G_{T}=\sum_{i=1}^{5} G_{i}$$

$$Fn_{T}=\sum_{i=1}^{5} \left( F_{i}\cdot n_{i} \right)$$

Substituting:

$$C_{w}=\frac{A}{G_{T}+Fn_{T}{\cdot q}_{r}}$$

To convert $C_{w}$ from $mg {ml}^{-1}$ to $\mu g L^{-1}$, let:

$$A^{'}= A\times{10}^{6}$$

Let $\Phi$ be a ‘fish consumption calibration factor’, i.e. the percentage difference in fish consumption of the ‘target population’ in comparison to the ‘baseline population’ (i.e. national average fish consumption). The following final equation is obtained:

$$\boldsymbol{C}_{\boldsymbol{w}}\left( \boldsymbol{\Phi} \right)\boldsymbol{[}\boldsymbol{\mu}\mathbf{g}\mathbf{L}^{\boldsymbol{-1}}\mathbf{]}\boldsymbol{=}\frac{\boldsymbol{A}^{\boldsymbol{'}}}{\boldsymbol{G}_{\boldsymbol{T}}\boldsymbol{+F}\boldsymbol{n}_{\boldsymbol{T}}\boldsymbol{\cdot}\boldsymbol{q}_{\boldsymbol{r}}\boldsymbol{\cdot\Phi}}\boldsymbol{, \Phi\in}\mathbb{R}_{\boldsymbol{\geq0}}$$

The domain and range of function $C_{w}(\Phi)$ is $[0,+\infty)$ and $(0, \frac{A^{'}}{G_{T}}]$, respectively. The function is associated with only one pharmaceutical because the variables are also bound to a specific pharmaceutical’s characteristics. This function can easily be applied after a HQ_t_ threshold is chosen and a Φ is established. Assuming HQ_t_ = 1, if $\Phi=0$, the lifetime consumption of fish by a ‘target population’ is zero times higher than the ‘baseline population’, then $C_{w}(0)=\frac{A^{'}}{G_{T}}$. This means the maximum acceptable $C_{w}$ is celled by the other exposure routes (dermal and oral via swimming, and oral via drinking water). There is a wider safety margin of $C_{w}(0)$, i.e. $0<C_{w}<\frac{A^{'}}{G_{T}}$. If $\Phi=2.5$, the lifetime consumption of fish by a ‘target population’ is 250% higher than the ‘baseline population’, then $C_{w}(2.5)=\frac{A^{'}}{G_{T}+Fn_{T}q_{r}\cdot2.5}$. This means a narrower safe margin of $C_{w}$, i.e. $0<C_{w}<\frac{A^{'}}{G_{T}+Fn_{T}q_{r}\cdot2.5}$. This is because naturally $\frac{A^{'}}{G_{T}+Fn_{T}q_{r}\cdot2.5}<\frac{A^{'}}{G_{T}}$. The higher the amount of fish consumed, the lower the concentration of contaminant should be allowed in surface waters inhabited by the fish. If $\Phi\to+\infty$, the lifetime consumption of fish by a ‘target population’ is infinitively higher than the ‘baseline population’, then $\lim_{\Phi\to+\infty} \left( C_{w} \right)=0$. This means water pollution has to be maintained extremely low levels, if the lifetime risk is to be kept below 1 (HQ_t_ < 1).
