# Supplementary figures and images for: Human health risk assessment of pharmaceuticals in the European Vecht River
Source: Integr Environ Assess Manag. 2022 Feb 28;18(6):1639–54. doi: 10.1002/ieam.4588 (PMC9790459; doi:10.1002/ieam.4588)

**GRAPHICAL ABSTRACT**

**
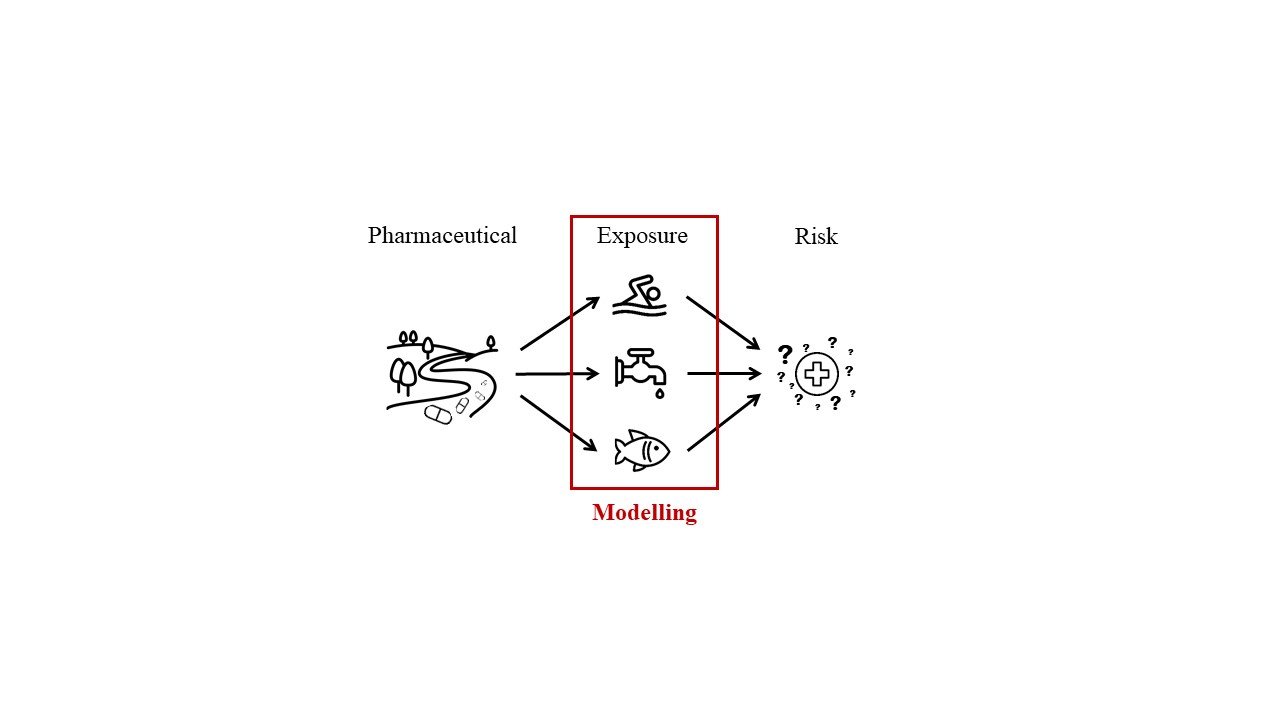
**

Supplement: Supplementary file 4 [file IEAM-18-1639-s004.docx]
